# Emerging Roles of SIRT5 in Metabolism, Cancer, and SARS-CoV-2 Infection

**DOI:** 10.3390/cells12060852

**Published:** 2023-03-09

**Authors:** Emanuele Fabbrizi, Francesco Fiorentino, Vincenzo Carafa, Lucia Altucci, Antonello Mai, Dante Rotili

**Affiliations:** 1 Department of Drug Chemistry and Technologies, Sapienza University of Rome, 00185 Rome, Italy; 2 Department of Precision Medicine, Università degli Studi della Campania “L. Vanvitelli”, 80138 Naples, Italy; 3 BIOGEM, 83031 Ariano Irpino, Italy; 4 IEOS—Istituto per l’Endocrinologia e Oncologia Sperimentale, CNR, 80131 Naples, Italy; 5 Pasteur Institute, Cenci-Bolognetti Foundation, Sapienza University of Rome, 00185 Rome, Italy

**Keywords:** SIRT5, sirtuins, HDACs, protein lysine desuccinylation, epigenetic, SIRT5 modulators

## Abstract

Sirtuin 5 (SIRT5) is a predominantly mitochondrial enzyme catalyzing the removal of glutaryl, succinyl, malonyl, and acetyl groups from lysine residues through a NAD^+^-dependent deacylase mechanism. SIRT5 is an important regulator of cellular homeostasis and modulates the activity of proteins involved in different metabolic pathways such as glycolysis, tricarboxylic acid (TCA) cycle, fatty acid oxidation, electron transport chain, generation of ketone bodies, nitrogenous waste management, and reactive oxygen species (ROS) detoxification. SIRT5 controls a wide range of aspects of myocardial energy metabolism and plays critical roles in heart physiology and stress responses. Moreover, SIRT5 has a protective function in the context of neurodegenerative diseases, while it acts as a context-dependent tumor promoter or suppressor. In addition, current research has demonstrated that SIRT5 is implicated in the SARS-CoV-2 infection, although opposing conclusions have been drawn in different studies. Here, we review the current knowledge on SIRT5 molecular actions under both healthy and diseased settings, as well as its functional effects on metabolic targets. Finally, we revise the potential of SIRT5 as a therapeutic target and provide an overview of the currently reported SIRT5 modulators, which include both activators and inhibitors.

## 1. Introduction

Lysine acylation, one of the most evolutionary conserved post-translational modifications (PTMs), is a reversible modification directed to nuclear proteins, such as histones, as well as mitochondrial and other non-nuclear proteins. Beyond acetylation, there is a heterogeneous pool of possible lysine acyl modifications, including fatty acylation, glutarylation, succinylation, malonylation, crotonylation, and 3-hydroxy-3-methylglutarylation [1,2]. Notably, protein acylation may proceed both enzymatically and spontaneously [3]. Recent evidence highlights that acyl-CoA thioesters, supported by the alkaline mitochondrial pH, can react spontaneously with lysine ε-amino groups. Dicarboxylic coenzyme A (CoA) thioesters with 4/5 carbon acyl backbones, such as succinyl-CoA and glutaryl-CoA, can react intramolecularly and create a high-energy cyclic anhydride, enhancing their reactivity towards nucleophilic lysine ε-amino groups [3,4]. Furthermore, although these CoA thioesters can difficultly pass through the mitochondrial membrane, different studies have shown that, thanks to coupled condensation/lysis mechanisms, these molecules can overcome this problem and move from the mitochondria to the cytosol. For example, acetyl-CoA reacts with oxalacetate to form citrate [5] that can freely diffuse through the nuclear pores [6]. In both the cytosol and the nucleus, citrate may be cleaved by the enzyme ATP-citrate lyase into oxalacetate and acetyl-CoA, which can acylate cytosolic and nuclear proteins [7,8]. These mechanisms lead to the accumulation of acylated proteins, which may cause the dysregulation of a variety of biochemical pathways, including glycolysis and fatty acid oxidation (FAO), among many others, finally causing an imbalance in the cellular metabolic equilibrium [3].

The enzymatic transfer of acetyl groups is catalyzed by lysine acetyltransferases (KATs) [9,10], while lysine deacetylases (KDACs) are enzymes whose primary function is to remove an acetyl residue, although they have been indicated to catalyze many deacylation reactions in different protein substrates but also in non-protein ones, such as polyamines [11,12,13]. KDACs are commonly divided into four classes. KDACs of classes I, II, IV are Zn^2+^-dependent deacylases characterized by structural analogies with reduced potassium dependency 3 (Rpd3) and histone deacetylase 1 (Hda1) in yeast [14]. Class III KDACs are nicotinamide adenine dinucleotide (NAD^+^)-dependent enzymes, also called sirtuins (SIRTs) because they share structural homology with the yeast silent information regulator 2 (Sir2) [15,16]. Differently from Zn^2+^-dependent KDACs, SIRTs require NAD^+^ as a catalytic cofactor, while the Zn^2+^ ion does not participate in the reaction mechanism and only has a structural role [17]. This unique NAD^+^-dependent mechanism has two important consequences. First, the catalytic activity of SIRTs is not limited to acetyl groups, but they are able to remove acyl groups such as myristoyl, palmitoyl, crotonyl, glutaryl, succinyl, and malonyl from the lysine ε-amino groups of histone and non-histone proteins [18]. Second, the requirement of SIRTs for NAD^+^ to conduct their activity makes them particularly sensitive to the cellular metabolic status. This is particularly relevant in all the situations in which there is a dysregulation of the NAD^+^/NADH ratio, such as in cases of malnutrition, obesity, carcinogenesis, and aging. Hence, SIRTs can be considered as biological sensors of the cellular metabolic status and their action may be affected by altered metabolic situations [17].

In mammals, seven SIRT isoforms have been discovered (SIRT1-7) [19], all of them possessing a conserved NAD^+^-binding domain and catalytic site, while they differ for the *N*- and *C*-termini which influence their subcellular localization and substrate specificity. SIRT1 is mainly located in the nucleus, along with SIRT6 and SIRT7 (specifically located in the nucleolus), even though it is also present in the cytosol [20,21,22,23]. SIRT2 is a cytoplasmatic protein, but it can move to the nucleus during mitosis. In addition, an alternate spliced version of SIRT2 has been observed as a constitutive nuclear protein [22,24,25]. SIRT3, 4, and 5 are mainly located in the mitochondria, which are essential for energy production, metabolism, and redox homeostasis. In line with this, SIRT3, SIRT4, and SIRT5 have been proposed to function as a link between metabolism and aging [20,26,27,28,29]. It is important to notice that SIRT3, 4, and 5 have multiple secondary localizations such as cytosol and nucleus, although in these compartments their concentration is lower than that in the mitochondria. Specifically, although primarily being a mitochondrial matrix protein, SIRT5 is also present in the cytosol, peroxisomes, and nucleus. Consistent with this, beyond mitochondrial proteins, several proteins in the cytosol and nucleus exhibit enhanced succinylation, malonylation, and glutarylation following SIRT5 loss [30,31,32,33,34].

Regarding their activity, SIRT1, SIRT2, and SIRT3 mostly show deacetylase activity [16]. SIRT4 and SIRT6 possess different activities, including mono ADP-ribosyltransferase, deacetylase, and deacylase activities, with SIRT4 also exhibiting lipoamidase activity [28,35,36,37,38]. In detail, SIRT4 can remove glutaryl, 3-methylglutaryl, 3-hydroxy-3-methyl-glutaryl (HMG), and 3-methylglutaconyl groups [39,40]. SIRT7 is involved in deacetylation, desuccinylation, and deglutarylation reactions [41,42].

**Figure 1:**
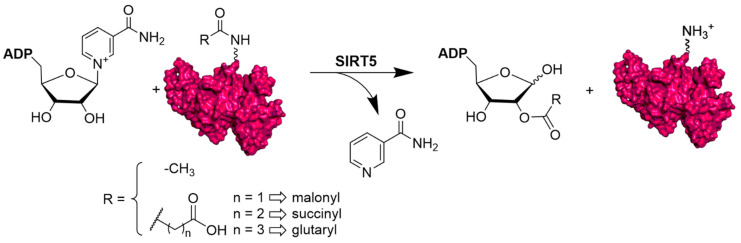
NAD^+^-dependent deacetylation, demalonylation, desuccinylation, and deglutarylation reactions catalyzed by human SIRT5.

Over the past years, thanks to the development of proteomics and the discovery of new PTMs, it has been possible to understand that SIRT5 is involved in different physiological functions. Indeed, SIRT5 exhibits a particular affinity for negatively charged acyl lysine modification and shows three main different activities: deglutarylation, desuccinylation, and demalonylation, along with a less efficient deacetylase activity (Figure 1) [19,43].

SIRT5, as well as all mitochondrial SIRTs, appears to be particularly involved in mitochondrial metabolism and cellular respiration [44]. Specifically, SIRT5 is involved in the regulation of glucose metabolism and glycolysis [31], FAO [12,45], amino acid degradation [44], and reactive oxygen species (ROS) homeostasis [46]. The roles played by SIRT5 in different pathways imply that its dysregulation is associated with the development of different diseases, including metabolic disorders, cardiovascular and neurodegenerative pathologies, infectious diseases, and cancer. Consequently, SIRT5 has gained interest as a possible drug target in the treatment of these diseases [47].

Here, we will examine the current knowledge about the structure and catalytic mechanism of SIRT5 and we will delve into its main targets and the biological, metabolic, and cancer-related functions associated with its dysregulation. Finally, we will report the most relevant compounds that modulate SIRT5 activity either as activators or inhibitors.

## 2. Structural and Functional Properties of SIRT5

**Figure 2:**
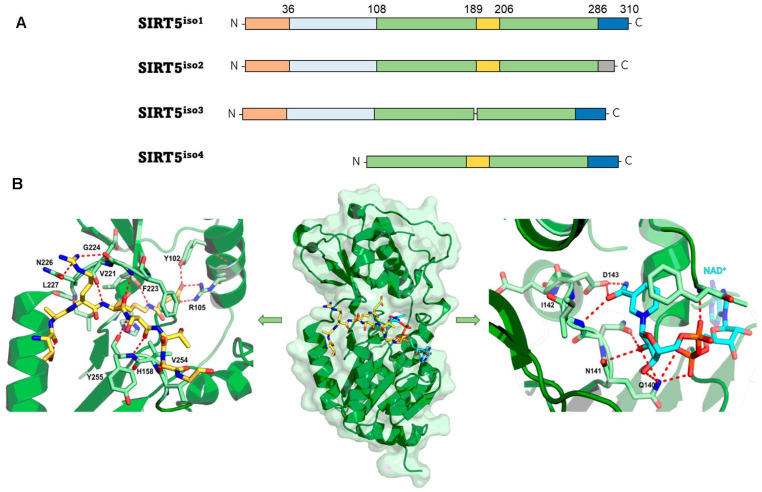
(**A**) The different SIRT5 isoforms. The N-terminal MLS is shown in orange; the 72-residue N-terminal portion present in SIRT5^iso1^, SIRT5^iso2^, and SIRT5^iso3^ is shown in light blue; the sequence common to all isoforms (108–188 and 207–286) is shown in green; the 18-residue sequence (189–206) lacking in SIRT5^iso3^ is shown in yellow; the C-terminal portion present in SIRT5^iso1^, SIRT5^iso3^, and SIRT5^iso4^ is shown in blue, while the different one present in SIRT5^iso2^ is shown in gray. (**B**) The structure of succinyl-H3K9 (green)/NAD^+^ (cyan) co-crystal (PDB code 3RIY). Left panel: zoomed view highlighting the interactions between SIRT5 and his substrate. Right panel: zoomed view of the SIRT5 catalytic pocket interacting with NAD^+^.

According to phylogenetic analysis, SIRT5 differs from other mammalian SIRTs and is part of the so-called class III sirtuin family, which primarily comprises prokaryotic SIRTs [48]. The SIRT5 gene produces four SIRT5 isoforms (SIRT5^iso1^ to SIRT5^iso4^) (Figure 2A). The *N*-termini of isoforms SIRT5^iso1^, SIRT5^iso2^, and SIRT5^iso3^ have a length of 72 amino acids plus 36-residue mitochondrial localization signal (MLS) peptide, but these sequences are lacking from SIRT5^iso4^. SIRT5^iso3^ lacks 18 amino acids corresponding to residues 189-206 of SIRT5^iso1^ and SIRT5^iso2^. SIRT5^iso1^, SIRT5^iso3^, and SIRT5^iso4^ have the same *C*-terminus (residues 286-310), while the *C*-terminal region of SIRT5^iso2^ (residues 286–299) is distinct from other isoforms in both length and sequence (Figure 2A). To date, the most well studied isoforms are SIRT^iso1^ and SIRT^iso2^, while SIRT^iso3^ and SIRT^iso4^ have been identified, but there is still no information about their localization and properties [49,50,51].

The resolution of the crystal structure of human SIRT5 (Figure 2B) indicated that SIRT5 presents 14 α-helices and nine β-strands and shares a central 268-residue region containing the catalytic and NAD^+^-binding sites with other SIRTs [52]. The structure of SIRT5 may be divided into two structural domains that share the substrate binding site: the Rossman-fold domain and the Zn^2+-^binding domain. The substrate binding site is defined by multiple loops connecting the two domains. Specifically, loop S, connecting α10 of the Rossman-fold domain with β6 of the Zn^2+^-binding domain, is pivotal for interaction with substrate. A large flexible loop, which ranges from Leu184 to Pro200 and connects α8 to α9, aids in achieving structural conformational changes following substrate binding [39,52,53,54,55,56,57]. NAD^+^ binding occurs at Loop N, which links α2 of the Rossman-fold domain and α3 of the Zn^2+^-binding domain (Figure 2B). This area contains several residues that are necessary for both substrate and co-substrate binding. The acyl-lysine substrate interacts directly with Ala86, Tyr102, Arg105, and His158 and Phe223, Leu227, and Val254 delineate the hydrophobic entrance for acyl-lysine (Figure 2B, left panel). Asp143 binds the nicotinamide product, whereas Gln140 and Asn141 engage in interactions with the ribose portion of NAD^+^ (Figure 2B, right panel). Additionally, Phe70 functions as a valve, enabling both the binding of NAD^+^ and the release of nicotinamide [19,52]. In SIRT1-3, some of these structural characteristics are retained [19,58,59,60]. For instance, in these orthologues, hydrophobic amino acids Phe223, Leu227, and Val254 are positioned in the same locations. SIRT5, on the other hand, has unique amino acids that define its substrate selectivity and enzymatic activity. Specifically, Tyr102 and Arg105 locate deep into the substrate binding site, where they interact with the negatively charged acyl-lysine substrate via hydrogen bonds and electrostatic interactions. The highly efficient deglutarylase, desuccinylase, and demalonylase activities of SIRT5 are provided by these residues, which are able to accommodate glutaryl, succinyl, and malonyl groups. Ala86 is a crucial residue for substrate interaction and is unique to SIRT5, since SIRT1–3 have instead a Phe residue in the same location. The acyl-lysine binding pocket of SIRT5 is bigger than those of other SIRTs due to the inclusion of alanine rather than phenylalanine, thereby allowing the entrance of bulkier acylated lysine substrates (Figure 2B) [52,61]. Like other SIRTs, SIRT5 also contains a structural Zn^2+-^binding domain made of five α-helices and three β-strands, thus forming an anti-parallel β-sheet. Here, four Cys residues (Cys166, Cys169, Cys207, and Cys212) coordinate the Zn^2+^ ion and contribute to maintain stable the antiparallel β-sheet (Figure 2B).

## 3. Biological Activities and Disease Relevance of SIRT5

### 3.1. Oxidative Stress

**Figure 3:**
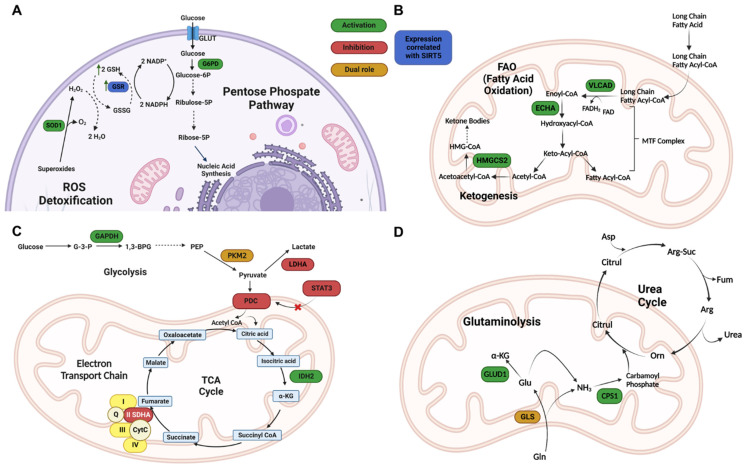
Redox homeostasis and metabolism regulation by SIRT5. (**A**) SIRT5 modulation of ROS detoxification enzymes. G6PD—glucose-6-phosphate dehydrogenase; GSH—reduced glutathione; GSR—glutathione reductase; GSSG—oxidized glutathione; Ribose-5P—ribose 5 phosphate; Ribulose-5P—ribulose 5 phosphate ROS, reactive oxygen species; SOD1—superoxide dismutase 1. (**B**) SIRT5 regulation of FAO enzymes. ECHA—enoyl-coenzyme A hydratase; HMGCS2—3-hydroxy-3-methylglutaryl CoA synthase 2; VLCAD—very long chain acyl-coenzyme A dehydrogenase. (**C**) SIRT5 roles in regulating glycolysis, TCA cycle, and ETC. 1,3-BPG—1,3-bisphosphoglycerate; α-KG—α-ketoglutarate; Acetyl-CoA—acetyl-coenzyme A; CytC—cytochrome C; G-3-P—glucose 3 phosphate; GAPDH—glyceraldehyde phosphate dehydrogenase; GLUT1—glucose transporter 1; IDH2—isocitrate dehydrogenase 2; LDHA—lactate dehydrogenase A; PDC—pyruvate dehydrogenase complex; PEP—phosphoenolpyruvate; PKM2—pyruvate kinase muscle isozyme 2; Q—Quinolone; SDHA—succinate dehydrogenase subunit A; STAT3—signal transducer and activator of transcription 3; TCA cycle—tricarboxylic acid cycle. (**D**) SIRT5 regulation of glutaminolysis and ammonia detoxification. α-KG—α-ketoglutarate; Arg—arginine; Arg-Suc—arginosuccinate; Asp—aspartate; Citrul—citrulline; CPS1—carbamoyl phosphate synthetase 1; Fum—fumarate; GDH—glutamate dehydrogenase; Gln—glutamine; GLS—glutaminase; Glu—glutamate; GLUD1—glutamate dehydrogenase 1; Orn—ornithine. Created with Biorender.com.

As SIRT5 is NAD^+^-dependent and the NAD^+^/NADH ratio is crucial for controlling oxidative stress, SIRT5 activity helps to maintain cellular redox homeostasis and regulate ROS levels (Figure 3A) [62]. The majority of cellular ROS are produced in the mitochondria because of oxidative metabolism. High ROS levels are detrimental to the cell because they can damage macromolecules and stimulate the intrinsic apoptosis pathway, whereas low ROS levels act as redox messengers [62,63]. Superoxide dismutases, enzymes that catalyze the conversion of superoxide into oxygen and hydrogen peroxide (which are then transformed into water by catalase or glutathione peroxidase), are among the protective detoxification mechanisms that cells have evolved as a result of their exposure to oxidative stress. In this context, SIRT5 overexpression has been demonstrated to increase SOD-mediated detoxification through desuccinylation of the active Cu/Zn superoxide dismutase (SOD1) [46]. Glutathione peroxidases, which utilize glutathione in its reduced form (GSH), are other enzymes able to detoxify water-derived peroxides. Glutathione that has been oxidized during the detoxification process (GSSG) is then reduced once again by glutathione disulfide reductase (GSR) which uses NADPH as a co-substrate. In this context, isocitrate dehydrogenase 2 (IDH2) and glucose-6-phosphate dehydrogenase (G6PD) are activated by SIRT5-mediated desuccinylation and deglutarylation, respectively, which results in enhanced NADPH synthesis and a corresponding decrease in ROS levels [46,64]. Additionally, glutathione disulfide reductase (GSR), an enzyme that reduces GSSG into GSH [65], was indicated to be hypo-expressed in SIRT5-deficient non-small cell lung cancer (NSCLC) cells, thus resulting in decreased detoxification activity and ROS level augmentation [66].

SIRT5 also deacetylates the forkhead protein FOXO3A, resulting in increased nuclear localization and consequent augmented expression of genes related to antioxidant activities [67]. Moreover, by preventing the dimerization of peroxisomal acyl-CoA oxidase 1 (ACOX1), a crucial enzyme implicated in the oxidation of fatty acids that produces H_2_O_2_ as byproduct, SIRT5 further contributes to reducing oxidative stress [63]. Overall, these reports point towards a crucial role of SIRT5 in defense mechanisms against oxidative stress.

### 3.2. Metabolism and Mitochondrial Regulation

SIRT5 regulates numerous metabolic pathways, including FAO (Figure 3B) [30,45], glycolysis [31], gluconeogenesis [31], the tricarboxylic acid (TCA) cycle, the electron transport chain (ETC) (Figure 3C) [44], and the urea cycle (Figure 3D) [68,69,70].

In the context of FAO (Figure 3B), the acyl-CoA dehydrogenase (ACAD) enzyme family dehydrogenates fatty acids into enoyl-CoA, which is converted into acetyl-CoA during the mitochondrial process known as β-oxidation. Three hypersuccinylated ACAD members have been identified in studies on the liver tissue of SIRT5-knockout (KO) mice: very long-chain acyl-CoA dehydrogenase (VLCAD), long-chain acyl-CoA dehydrogenase (LCAD), and medium-chain acyl-CoA dehydrogenase (MCAD) [45,71]. In particular, it was shown that muscles and liver from SIRT5-KO mice accumulated medium- and long-chain acyl-carnitines and that SIRT5, in conjunction with SIRT3, promotes their localization in the mitochondrial membrane via desuccinylation and deacetylation, respectively. These activities also facilitate the interaction between VLCAD and its cofactor flavin adenine dinucleotide (FAD), thereby promoting its activity [45].

SIRT5 has recently been demonstrated to desuccinylate and activate enoyl-CoA hydratase (ECHA) in mouse myocardium [45,72,73], thereby promoting FAO. SIRT5-deficient hearts also show poor fatty acid metabolism and reduced ATP synthesis during fasting and exercise [33]. In addition, SIRT5 contributes to the regulation of ketone body synthesis by desuccinylating and consequently increasing the activity of HMG CoA synthase 2 (HMGCS2) (Figure 3B) [32].

Several studies have indicated that SIRT5 is implicated in the modulation of many enzymes involved in the glycolytic process (Figure 3C). Nishida and colleagues showed that SIRT5 demalonylates glyceraldehyde 3-phosphate dehydrogenase (GAPDH) and different glycolytic enzymes, thus promoting glycolysis [31]. This is demonstrated by the decreased glycolytic activity in the hepatocytes of SIRT5 KO mice [31]. Furthermore, SIRT5 activity is implicated in insulin sensitivity and adipose tissues are characterized by elevated SIRT5 expression and associated with optimal response to insulin [74]. Interestingly, a recent study found that SIRT5 is overexpressed in the kidney cortex of type 2 diabetic BKS *db/db* mice. This was associated with reduced malonylation of enzymes involved in glycolysis and peroxisomal FAO, which in turn promotes both processes [75]. Experiments in cultured human kidney proximal tubules (HK-2 cells) revealed that higher SIRT5 expression is associated with increased glycolysis and reduced entry of pyruvate into the TCA cycle in low glucose conditions. Differently, in high glucose conditions, the authors found augmented levels of TCA metabolites. In line with these observations, SIRT5 was found overexpressed in the kidneys of type 2 diabetes patients [75].

Lys311 desuccinylation of pyruvate kinase M2 (PKM2), one of the glycolytic enzymes controlled by SIRT5, was found to promote glycolytic activity. PKM2, which converts phosphoenolpyruvate into pyruvate, occurs in two distinct quaternary structures: as a tetramer, it has significant pyruvate kinase activity, though as a dimer, which is mostly found in the nucleus, it primarily functions as a protein kinase [76,77,78,79]. Interestingly, loss of SIRT5 in lipopolysaccharide (LPS)-activated macrophages causes the switch of cellular metabolism towards glycolysis, despite the evidence that SIRT5 is implicated in the promotion of glycolytic activity [79]. Furthermore, according to Xiangyun et al., desuccinylation of PKM2 Lys498 under oxidative stress decreases its activity in cancer cells and slows glycolysis by causing a transition from the glycolytic route to the pentose phosphate pathway [80]. These two findings demonstrate that the role of SIRT5 in glycolysis is context-dependent. Another study found that PKM2 desuccinylation during glucose deficiency prevented its translocation in the mitochondria and facilitated voltage dependent anion channel 3 (VDAC3) degradation by promoting mitochondrial pore opening, higher permeability, and inducing apoptosis in colon cancer cells [81].

The pyruvate dehydrogenase complex (PDC), which is involved in pyruvate oxidation to acetyl-CoA, is also controlled by SIRT5 [82]. Indeed, SIRT5 was shown to deacetylate the signal transducer and activator of transcription 3 (STAT3), reducing its binding and consequent activation of PDC as well as decreasing its mitochondrial translocation. As a result, oxidation of pyruvate into acetyl-CoA is impaired, and acetyl-CoA cannot enter the TCA cycle [74]. Moreover, SIRT3 was shown to contribute to STAT3 deacetylation, albeit to a much smaller extent. Nevertheless, the biological implications of SIRT3-mediated deacetylation of STAT3 were not discussed. Furthermore, the impact of SIRT5 on STAT3 deacylation (such as desuccinylation) was not evaluated. Therefore, given the weak deacetylase activity of SIRT5, we may not rule out the possibility that it also functions as a STAT3 desuccinylase [82]. In line with these results, SIRT5-KO cells were shown to possess enhanced PDC activity, which results in higher pyruvate-dependent cellular respiration. The finding that SIRT5 ablation results in enhanced ATP synthesis confirms that SIRT5 inhibition of PDC causes an imbalance in pyruvate metabolism with a loss in ATP generation [30]. Again, the context specificity of SIRT5 is shown by the fact that pyruvate-driven respiration is decreased in SIRT5-deficient HEK293 cells whereas ATP synthesis and oxygen consumption are increased in HepG2 cells when SIRT5 is overexpressed [83].

Through its desuccinylase activity, SIRT5 also modulates TCA enzymes (Figure 3C). SIRT5 desuccinylates and activates IDH2, an enzyme catalyzing the NADP^+^-dependent oxidative decarboxylation of isocitrate to α-ketoglutarate (α-KG), producing CO_2_ and NADPH [64,84]. Differently, Park et al. suggested that SIRT5-mediated desuccinylation impairs the activity of succinate dehydrogenase (SDH, also known as Complex II), which is a component of both TCA and ETC and catalyzes the oxidation of succinate to fumarate and the conversion of ubiquinone to ubiquinol. Consistent with this, SIRT5 knockdown (KD) caused an increase in SDH activity, which is connected to an increase in succinate-dependent respiration [30]. Additionally, following interaction with cardiolipin, SIRT5 promotes the function of the respiratory chain by desuccinylating proteins located in the inner mitochondrial membrane, such as the members of all four ETC complexes and ATP synthase [83]. As a result, SDH and ATP synthase enzyme activities are hindered in SIRT5-KO liver homogenates, and SDH-driven respiration is decreased [85], in contrast with the previous study by Park and colleagues. Additionally, SIRT5 interacts with cytochrome C and the Complex I component NDUFA4, although its exact role has not been determined yet [85].

SIRT5 activity also influences the regulation of nitrogenous waste (Figure 3D). There are many detoxification systems that convert ammonia into urea through the urea cycle (also called ornithine cycle), which occurs mainly in the liver. Nitrogenous waste products, such as uric acid and ammonia, created during nucleic acid and protein degradation may be hazardous if they accumulate in cells. Carbamoyl phosphate synthetase 1 (CPS1), an enzyme involved in the conversion of ammonia and bicarbonate into carbamoyl phosphate, is deacylated and activated by SIRT5 in liver cells [52,68,86,87]. In line with this, Nakagawa et al. found that SIRT5-KO mice show lower CPS1 activity and higher blood ammonia levels during high amino acid catabolism settings [68]. Ogura et al. highlighted that overexpression of SIRT5 in mouse models augmented hepatic CSP1 activity and that during caloric restriction (CR) mouse livers have higher SIRT5 mRNA expression. These results indicate that SIRT5 may have a crucial function in the metabolic adaptation to CR [87].

Two studies have shown conflicting results about how SIRT5 affects the synthesis of ammonia in non-liver cells, where it was shown to desuccinylate mitochondrial glutaminase (GLS) [69,70]. Polletta et al. showed that GLS activity is inhibited by SIRT5-mediated desuccinylation, which in turn suppresses glutamine conversion into glutamate and the consequent production of ammonia. Lys245 and Lys320 were suggested by the authors as potential sites of succinylation that could be reached by SIRT5. Since ammonia can trigger both mitophagy and autophagy in cancer cells, SIRT5-mediated suppression of GLS could circumvent this defense mechanism, pointing to an oncosuppressor function for SIRT5 in this setting [69]. On the other hand, another report found that Lys 164 by SIRT5 stabilizes GLS and boosts glutamine catabolism by preventing GLS from being ubiquitinated at Lys164 and then degraded by the proteasome [70].

Additionally, SIRT5 is involved in thermogenesis and is expressed in brown adipose tissue (BAT). Specifically, SIRT5 was shown to demalonylate and desuccinylate several proteins, such as uncoupling protein 1 (UCP-1) [88,89,90,91]. In a mouse model, SIRT5 KD causes proteins to be highly succinylated and lowers the activity of UCP-1. This leads to a drop in α-KG levels and an increase of the repressive histone marks H3K9me2/3 at the promoter of *Prdm16*, a transcription factor that regulates the expression of brown adipocyte genes [92]. Overall, SIRT5 is essential for the activation of brown adipogenic genes and contributes to the differentiation of brown adipocytes and the conversion of white adipose tissue (WAT) into BAT [92]. Because SIRT5 is essential in preserving BAT/WAT balance, and since BAT is involved in glucose homeostasis, SIRT5 may be a target for the therapy of certain metabolic diseases such as obesity and type 2 diabetes [93].

Beyond regulating metabolism, SIRT5 controls a variety of mitochondrial functions, including elongation, fusion, and division. In line with this, under starving conditions, dynamin-related protein 1 (DRP1) levels increased and mitochondrial fragmentation and mitophagy were amplified in SIRT5-KO mouse embryonic fibroblasts (MEFs). This demonstrates that SIRT5 protects mitochondria against autophagy and degradation brought on by starvation [94]. Moreover, SIRT5 activity exerts a protective role in the context of intervertebral disc degeneration (IDD). Under mechanical stress, SIRT5 overexpression significantly decreased apoptosis in nucleus pulposus (NP) cells [95]. Conversely, SIRT5 KD enhanced apoptosis and NP cell dysfunction, and SIRT5 KO mice exhibited a pronounced IDD phenotype. The authors showed that SIRT5 desuccinylates the apoptosis inducing factor mitochondrion-associated 1 (AIFM1). As a result, SIRT5 KD enhances AIFM1 succinylation while decreasing its interaction with CHCHD4, a mitochondrial protein involved in the import and folding of small cysteine-containing proteins in the mitochondrial intermembrane space, including those implicated in the ETC. Since the interaction between AIFM1 and CHCHD4 is essential for the biogenesis of respiratory chain complexes, its disruption results in decreased ETC and subsequent mitochondrial dysfunction, which accelerates the development of IDD under mechanical stress. In line with these findings, SIRT5 overexpression in a compression-induced rat IDD model could reverse mechanical stress-induced damage [95].

### 3.3. Cardiovascular Regulation

The effects of SIRT5 on cardiac activity have been the subject of numerous studies. As previously stated, ECHA, an enzyme pivotal for fatty acid catabolism in the myocardium, is activated by SIRT5-mediated desuccinylation. Consistent with this, SIRT5 KO reduces FAO and energy production in the heart under exercise or during fasting and leads to hypertrophic cardiomyopathy [33].

The comparison of transgenic SIRT5-overexpressing, SIRT5 KO, and wild-type mice in which cardiac hypertrophy and heart failure (HF) were induced by transverse aortic constriction (TAC) has revealed that an increase in SIRT5 deacylase activity is associated with an improvement in cardiac function and a decrease in fibrosis during pressure overload [96]. Conversely, a higher propensity for cardiac ischemia–reperfusion damage has been linked to SIRT5 deficiency [96]. In addition, WT TAC mice exhibited overexpression of glycolytic genes and downregulation of genes involved in fatty acid catabolism and oxidative phosphorylation [96]. According to this study, SIRT5 affects immune system infiltration after TAC, late LV remodeling and dysfunction, cardiac fibrosis, ventricular dilatation, and T cell expansion through modulating the inflammatory response. These effects are suggested to be driven by desuccinylation and activation of PKM2 [96]. Overall, the authors suggest that SIRT5 may be involved in controlling cytokine-mediated fibrosis activation, immune cells that produce cytokines, fibroblast activation, and/or myofibroblasts themselves [96].

SIRT5 KO in cardiac tissue leads to elevated levels of succinylated lysine proteins [33,34], including SDH, whose activity is impaired following desuccinylation [30]. Consistent with this, SIRT5-KO hearts treated with dimethyl malonate, a precursor of malonate, an SDH inhibitor, resulted in a lower production of superoxide, demonstrating the critical role of SIRT5 in controlling ROS production at the cardiac level [97]. This was supported by a different study that demonstrated that SDH inhibition in the heart protects against myocardial ischemia–reperfusion injury [98].

Finally, by a not-specified interaction with Bcl-XL, an anti-apoptotic member of the Bcl-2 family, SIRT5 was indicated to prevent H_2_O_2_-driven apoptosis in cardiomyocytes [99].

### 3.4. Neurodegeneration

Energy production, apoptosis, redox homeostasis, and ROS level regulation are crucial for maintaining the health of neurons. In fact, changes to these processes have a role in the development of neurodegenerative diseases such as Parkinson’s disease (PD), Alzheimer’s disease (AD), dementia, and epilepsy disorders [100]. Numerous investigations have indicated that SIRT5 has a neuroprotective effect because of its ROS detoxification activity.

1-methyl-4-phenyl-1,2,3,6-tetrahydropyridine (MPTP) is a convulsant used to induce PD symptoms in animals. This compound is converted into 1-methyl-4-penylpyridinium (MPP+) in vivo, which results in the degeneration of dopaminergic neurons of the substantia nigra in mice, increases levels of ROS, and causes cell death [101,102]. Interestingly, mice treated with MPTP display higher levels of SIRT5 in their brains. Consistent with this, a SIRT5 deficit in mouse brain striates accelerated the death of nigrostriatal dopaminergic neurons brought on by MPTP. Lower expression of the mitochondrial enzyme manganese superoxide dismutase 2 (SOD2) was linked to this [66]. These findings imply that SIRT5 activity mitigates the negative effects of MPTP and promotes ROS scavenging in nigrostriatal dopaminergic neurons.

Several studies have demonstrated that the activity of SIRT5 mitigates neuronal injury by lowering oxidative stress and the activity of astrocytes and microglia, demonstrating its protective effect against AD. SIRT5 downregulation and decreased autophagy were observed in AD mouse models, and these effects could be overcome by SIRT5 overexpression [103]. Additionally, SIRT5 expression, both in vitro and in vivo, was linked to increased SOD activity, decreased ROS levels, and reduced apoptosis. Moreover, AD brains expressing high amounts of SIRT5 display lower neuronal damage and inflammation, which may be due to microglia and astrocyte activation [103].

Other studies have demonstrated that SIRT5 protects against epileptogenic disorders [104]. When mice were exposed to kainate, a glutamate analogue with epileptogenic and neuroexcitatory activity, SIRT5 levels augmented in the hippocampus, highlighting its neuroprotective role against the development of astrogliosis. Conversely, SIRT5 KO causes a strong epileptogenic response in kainate-exposed animals [104,105]. Interestingly, the protective action of SIRT5 in this situation is not linked to its activity in ROS detoxification.

### 3.5. Inflammation

Recent evidence also points towards a role for SIRT5 in the modulation of the inflammatory response. In the context of the chronic inflammatory skin disease psoriasis, SIRT5 was indicated to have a protective function by decreasing keratinocyte proliferation and the production of inflammatory proteins [106]. The primary cause of psoriasis is excessive keratinocyte proliferation; STAT3 can promote both cell growth and differentiation. Moreover, psoriasis is characterized by an epidermal barrier dysfunction, which is promoted by the ERK/STAT3 signaling pathway. Overexpression of SIRT5 in interleukin-17A (IL-17A)-stimulated keratinocytes was shown to reduce the levels of p-ERK and STAT3. In addition, SIRT5 expression was positively correlated with the levels of fatty acid elongase 1 and 4 (ELOVL1 and ELOVL4, respectively), filaggrin, loricrin, and aquaporin-3, which are factors that contribute to maintaining normal barrier function [106]. Similarly, in the context of septic acute kidney injury, SIRT5 overexpression was associated with higher levels of phosphorylated AMPK and could alleviate mitochondrial dysfunction in renal tubular epithelial cells. This resulted in the reduction of mitochondrial structural damage, the recovery of ATP production, and the decrease of pro-apoptotic protein expression and ROS production. Overall, this study demonstrates that SIRT5 can reduce septic acute kidney injury [107].

Conversely, SIRT5 was shown to promote neuroinflammation and its expression was indicated to increase following ischemic stroke **[108]**. Mechanistically, SIRT5 desuccinylates annexin A1 (ANXA1) at Lys166, which in turn leads to a decrease in its SUMOylation. Desuccinylation of ANXA1 enhances nuclear localization while inhibiting membrane recruitment and extracellular secretion. ANXA1 is a key regulator of microglia-induced inflammation and its role is greatly reliant on its subcellular localization. Indeed, ANXA1 reduces microglial aberrant activation when delivered to the plasma membrane. In line with this, SIRT5 overexpression significantly upregulated the mRNAs of proinflammatory proteins (*Cxcl1*, *Ccl2*, *TNF-α*, *IL-6*, and *IL-1β*) as well as the protein levels of inducible nitric oxide synthase (iNOS), CD16/32, and Iba-1. Altogether, these factors contribute to neuronal injury. In line with this, SIRT5 KD in the microglia of middle cerebral artery occlusion (MCAO) mouse models exerted a protective role against cerebral ischemia/reperfusion injury [108].

### 3.6. Cancer: A Janus-Faced Role

**Figure 4:**
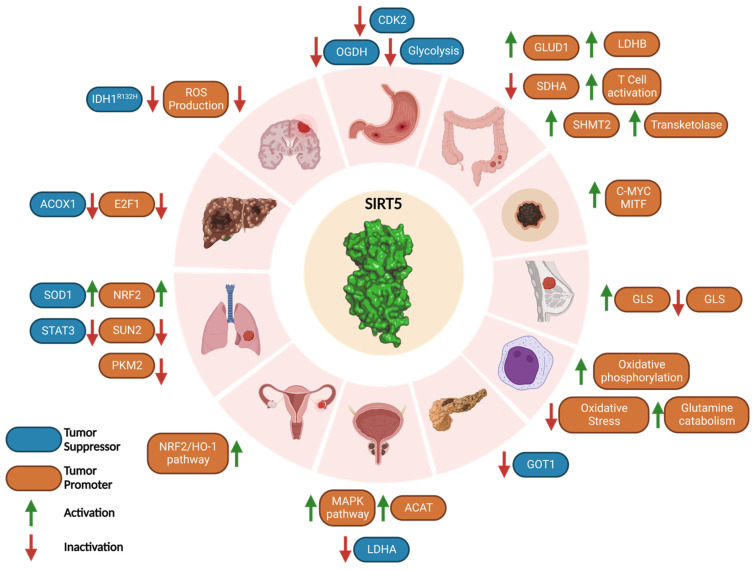
SIRT5 roles in carcinogenesis. SIRT5 plays a Janus-faced role acting as either tumor promoter or suppressor in a context-dependent manner. The types of cancer reported in this scheme are, in clockwise order, gastric cancer, CRC, melanoma, breast cancer, AML, PDAC, prostate cancer, ovarian cancer, lung cancer, HCC, medulloblastoma, and glioma. Created with Biorender.com.

Like other SIRTs, the role of SIRT5 in cancer is rather controversial [16,38,47,109,110]. In humans, the SIRT5 gene is present in the highly unstable cytogenetic band on chromosome 6p23 [49,111]. The SIRT5 gene locus is commonly gained or lost in a variety of cancers, but typically in the context of non-focal genomic events that modify many neighboring genes in addition to SIRT5 [49,111,112,113]. Although there is evidence of SIRT5 mRNA expression in a range of malignancies, many studies demonstrated that SIRT5 may act as either a tumor suppressor or a tumor promoter, depending on the specific pathways it regulates. In particular, SIRT5 mRNA levels are increased in some cancers such as NSCLC, colorectal cancer (CRC), and Waldenstrom’s macroglobulinemia [66,114,115], while they are significantly decreased in others, including endometrial carcinoma and head and neck squamous cell carcinoma (HNSCC) [63,116,117]. Moreover, SIRT5 has a dichotomous function in lung cancer [46,66,82,118], hepatocellular carcinoma (HCC) [63,119,120,121], and breast cancer [69,70], demonstrating that its activity ultimately depends on the particular environment and not only on the kind of tissue or cancer type. Taken together, these studies suggest that SIRT5 has a context-dependent role in each mutation-specific cancer subtype (Figure 4).

#### 3.6.1. Tumor Promoter Role of SIRT5 in Cancer

By interfering with many pathways, SIRT5 acts as a tumor promoter in a variety of malignancies. It has been noted that SIRT5 promotes carcinogenesis and cell proliferation and is overexpressed in breast cancer. As mentioned before, Greene et al. highlighted that SIRT5 protects GLS from ubiquitination and consequent proteasomal degradation, increasing glutamine catabolism and producing α-KG, which contributes to ATP production since it is part of the TCA cycle, finally supporting cancer cell proliferation [70]. Higher SIRT5 levels in breast cancer patients were associated with a poor prognosis. In line with this, SIRT5 suppression severely reduced cell proliferation in MCF7 and MDA-MB-231 breast cancer cells [70]. Moreover, SIRT5 inhibition arrested proliferation and the anchorage-independent growth of MCF7 and MDA-MB-231 breast cancer cells [122].

In cutaneous and uveal melanoma, SIRT5 performs a proto-oncogenic function and CRISPR/Cas9-mediated depletion causes significant loss of cellular viability and activation of apoptosis [123]. Specifically, SIRT5 promotes cancer development in both *Braf/Pten*-driven autochthonous melanoma and xenograft mouse models of melanoma. Transcriptomic investigations show that SIRT5 is essential for melanoma cells to maintain balanced gene expression. Notably, *c-Myc* and the lineage-specific oncogenic transcription factor *MITF* are SIRT5-dependent genes and it has been observed that SIRT5 activity may promote their expression by sustaining histone acetylation and methylation at their promoters [123]. Specifically, MITF is a member of the microphthalmia family of transcription factors and is dysregulated in melanoma. Humans and mice lacking MITF activity show reduced melanocyte development and pigmentation, demonstrating the critical role of MITF in melanocyte survival and function. MITF amplification is seen in 15% to 20% of melanomas and is linked to a poor prognosis. It is also known to play important roles in melanoma cell survival and differentiation [123].

SIRT5 was also found overexpressed in cultured SH-EP neuroblastoma cells and protected them from apoptosis by lowering ROS levels, thereby exerting a tumor promoting action. [124].

It has been shown that SIRT5 is highly expressed in HCC cell lines and that SIRT5-depleted cells exhibit a high apoptotic index, reduced invasion, and reduced cell proliferation in vitro. In this context, the E2F1 transcription factor, which controls the cell cycle by promoting cell proliferation, is positively regulated by SIRT5, thereby facilitating cancer cell proliferation and invasion [121,122].

Compared with normal tissues, ovarian cancer displays higher levels of SIRT5 [125]. In this setting, by controlling the NRF2/HO-1 pathway, which contributes to raising GSH levels, SIRT5 confers resistance to genotoxic chemotherapeutics such as cisplatin, thereby supporting tumor growth [126].

Yang and colleagues found in a recent study that serine hydroxymethyltransferase 2 (SHMT2) desuccinylation on Lys280 by SIRT5 leads to an increase in its enzymatic activity and tumor cell proliferation. SHMT2 is engaged in the metabolism of folate and catalyzes the conversion of serine and tetrahydrofolate (THF) into glycine and 5,10-methylenetetrahydrofolate (5,10-CH_2_-THF), an important stage in the biosynthesis of the purine nucleotides, which is crucial for sustaining cancer cell proliferation [127]. Notably, hypersuccinylation of SHMT2 caused by SIRT5 KO in osteosarcoma (U2OS) and CRC (HCT116) cells or expression of succinylation mimic mutant (K280E) significantly reduced cancer cell growth both in vitro and in vivo [128].

Recent studies found that expression of SIRT5 in CRC cells and tissue is linked to poor prognosis [129] and that SIRT5 KD in CRC cells (HCT116 and LoVo) leads to reduced proliferation [130]. Mechanistically, SIRT5 was shown to deglutarylate and activate glutamate dehydrogenase 1 (GLUD1), which increases the production of α-KG, thereby supporting CRC cell growth [130]. Additionally, several studies have demonstrated a link between SIRT5 and chemoresistance in CRC. In particular, CRC cells expressing wild-type KRas and SIRT5 exhibited resistance to various chemotherapeutics and antibody-based medications such as the EGFR inhibitor cetuximab. In line with this, in patients with wild-type *KRas* CRC, increased expression of SIRT5 is linked to a shorter time to post-therapy recurrence and a generally unfavorable prognosis [131]. Additionally, according to Du et al., SIRT5 is able to demalonylate and inactivate the SDH subunit A (SDHA), which leads to succinate accumulation and activation of thioredoxin reductase 2 (TrxR2), a ROS scavenger that is crucial for maintaining redox homeostasis and a key factor in chemotherapeutic resistance [131]. Furthermore, the α-KG dependent dioxygenases, which are proteins involved in histone and DNA/RNA demethylation, are inhibited by succinate and the consequent epigenetic dysregulation promotes carcinogenesis and the emergence of cetuximab resistance [131]. In CRC cells, SIRT5 was recently indicated to demalonylate and consequently activate transketolase, an enzyme involved in the non-oxidative pentose phosphate pathway, thus promoting the production of ribose-5-phosphate, an essential precursor of nucleotides [132]. This activity protects CRC cells from DNA damage by sustaining the nucleotide pool. Indeed, SIRT5 KD in CRC cells induced substantial DNA damage and a reduction of the levels of purine nucleotides. This was associated with cell cycle arrest and apoptosis, as well as significant inhibition of transketolase. Moreover, similar observations were made following treatment of CRC cells with the SIRT5 inhibitor **8d** (see Section 4.2) [132].

SIRT5 was also shown to promote autophagy through deacetylation and activation of lactate dehydrogenase B (LDHB), which converts lactate into pyruvate using NAD^+^ as a co-substrate, thereby yielding NADH and H^+^, which cause lysosomal acidification and trigger autophagy [129]. In addition to being a crucial autophagy regulator, LDHB converts lactate to pyruvate, which powers the TCA cycle and helps oxidative cancer cells. In HCT116 cells, LDHB K329 deacetylation performed by SIRT5 supported cell respiration and ATP synthesis, thereby promoting the growth of cancer cells also through this pathway. In line with this, SIRT5 KO or LDHB KD reduced CRC cell growth, and cancer cells overexpressing wild-type LDHB expanded more quickly than those overexpressing LDHB-K329Q. Additionally, it was shown that the LDHB K329 acetylation in CRC tissues was lower than in healthy ones and was associated with a poor prognosis for CRC patients. The LDHB-Ac-K329 status is therefore a possible prognostic factor for CRC patients and may be beneficial for identifying the CRC patients who are suited for anti-autophagy therapy [129].

Recent research has demonstrated the connection between SIRT5 function, T cell activation and differentiation, and CRC growth. Indeed, proteomics experiments indicated that the expression of several proteins involved in the T cell receptor signaling pathway differs between SIRT5 KO and wild-type T cells. Specifically, SIRT5 KO enhances IFN-β production, which in turn affects T cell development by promoting naive T cell activation, increases T-helper 1 (Th1) and cell toxicity T lymphocytes (CTL) differentiation, and decreases CD4+ regulatory T (Treg) cell differentiation. This imbalance between the inflammatory Th1 and the immunosuppressive Treg cells finally leads to cancer cell proliferation [133].

SIRT5 was shown to be overexpressed in advanced NSCLC and linked to poor prognosis in a study by Lu et al. that highlighted the possible function of SIRT5 in promoting lung cancer development and drug resistance [66]. The fact that SIRT5 stimulates the production of the transcription factor NRF2 is unquestionably one of the major contributing elements to NSCLC development. NRF2 controls the expression of several genes involved in oxidative stress defense and xenobiotic tolerance. In line with this, SIRT5 KD cells exhibit lower levels of NRF2 and its target genes, increasing their vulnerability to genotoxic chemotherapeutics (cisplatin, 5-fluorouracil, and bleomycin) [66]. Another study revealed that SIRT5 negatively modulates the expression of SAD1/UNC84 domain protein 2 (SUN2), a crucial subunit of the linker of nucleoskeleton and cytoskeleton (LINC) complex [118]. SUN2 acts as a tumor suppressor since its activity counteracts the Warburg effect, a metabolic change in which glycolysis predominates over oxidative phosphorylation as the major source of ATP, providing rapid energy to drive cancer cell growth. In line with this, SUN2 overexpression decreases lung cancer cell proliferation and migration and makes cancer cells more susceptible to cisplatin-induced apoptosis. In this context, SIRT5 inhibits the expression of SUN2, thereby promoting the Warburg metabolism switch; this is associated with a poor patient prognosis [118].

As previously mentioned, SIRT5 desuccinylates PKM2, although two studies suggested different desuccinylation sites (Lys498 or Lys311) [79,80]. Independently from the desuccinylation sites, both studies provide evidence that SIRT5 suppresses PKM2 kinase pyruvate activity and that this has a proto-oncogenic impact. Indeed, the accumulation of glycolytic intermediates caused by PKM2 inhibition promotes the pentose phosphate pathway, which increases NADPH levels and supports tumor development. In line with this, A549 cells treated with the SIRT5 inhibitor suramin were characterized by an increase in PKM2 activity and a reduction of cell proliferation [80]. Moreover, SIRT5 inhibition had no impact in A549 cells where wild-type PKM2 was replaced with the tumor-suppressing succinylation mimetic mutant K489E, bearing a negatively charged glutamate in place of the lysine residue targeted by SIRT5 [80].

SIRT5 is also significantly expressed in prostate cancer, where it activates acetyl-CoA acetyltransferase 1 (ACAT1) and induces a mitogen-activated protein kinase (MAPK) pathway. The MAPK pathway alters the expression of matrix metallopeptidase 9 and cyclin D1 activity, which promote prostate cancer cell migration and proliferation [134].

SIRT5 also fosters the development of acute myeloid leukemia (AML). The potential of SIRT5 to promote the survival of tumor cells by lowering oxidative stress and maintaining glutamine catabolism and oxidative phosphorylation [135] is linked to its tumor-promoter action. Studies using syngenic and xenograft mouse models of AML have shown that overexpression of SIRT5 causes tumorigenesis and cancer growth. In addition, SIRT5-KD AML cells demonstrate a significant propensity for apoptosis as well as decreased proliferation and colony formation. Similar effects have been observed in AML cell cultures (OCI-AML2, SKM1, and MOLM-13) treated with SIRT5 inhibitors (see compounds **8b**, **8d**, and **8i** in Section 4.2.2) [135,136].

Finally, a recent study found that SIRT5 inhibits the tumor suppressor p53 by desuccinylating its Lys120, which suppresses its activation and results in a decrease in the transcription of p53-related genes and apoptosis. These findings demonstrate how SIRT5 promotes cancer development by inhibiting the activities of the tumor suppressor p53 in several cancer types [137].

#### 3.6.2. Tumor Suppressor Role of SIRT5 in Cancer

SIRT5 also exhibits a tumor suppressor behavior in a variety of cancer types by interfering with several pathways. In the next paragraphs, we will also discuss its oncosuppressor role in some of the cancer types mentioned in the previous section, which emphasizes that the functional role of SIRT5 varies depending on both the tissue type and the specific setting.

SIRT5 functions as an oncosuppressor in pancreatic ductal adenocarcinoma (PDAC), according to a recent study by Hu et al. [138]. Indeed, SIRT5 is downregulated in murine pancreatic tumors and human PDAC tissues, and its hypoexpression has been linked to cancer development and poor prognosis. KRas-mutated PDAC cells metabolize glutamine via the GOT2/GOT1/ME1 pathway, which is not required by other cells. In this context, SIRT5 deacetylates Lys369 of the aspartate aminotransferase GOT1 and impairs its function. GOT1 converts aspartate and α-KG into glutamate and oxalacetate in the cytosol, thereby raising the concentrations of NADPH and GSH required to maintain redox equilibrium and promoting PDAC cell proliferation. Hence, GOT1-mediated SIRT5 suppression results in decreased cancer cell detoxification systems, an increase in ROS, and decreased proliferation. On the other hand, SIRT5 depletion reduces ROS levels and promotes the growth of cancer cells [138].

SIRT5 seems to arrest gastric cancer cell growth by interfering with two separate routes [139]. Indeed, SIRT5 blocks the cell cycle of cancer cells at the G1/S phase by negatively modulating cyclin dependent kinase 2 (CDK2) and inhibiting glycolysis. Furthermore, overexpression of SIRT5 inhibits oxoglutarate dehydrogenase (OGDH), which in turn lowers ATP levels and raises ROS levels, thereby impairing the proliferation and migration of cancer cells [140].

Studies conducted in vitro and in vivo have demonstrated that the desuccinylase activity of SIRT5 is essential for preserving mitochondrial activities and inhibiting cell growth in the case of glioma [44]. *IDH1* and *IDH2* mutations have been found in glioma, chondrosarcoma, and AML, according to research by Clark and colleagues [141]. α-KG is converted into *R*-2-hydroxyglutarate by IDH1 and IDH2 mutants instead of being converted into isocitrate [84,142]. *R*-2-hydroxyglutarate inhibits SDH and α-KG-dependent dioxygenases and in turn raises the levels of succinyl-CoA and causes abnormal succinylation of mitochondrial proteins, which promotes the growth of cancer cells and impairs apoptosis [44,143]. In addition, in glioma cells characterized by the presence of the IDH1-R132H mutant, hypersuccinylation causes an upsurge in Bcl-2 levels with consequent apoptosis resistance. Hence, SIRT5 overexpression results in lower amounts of protein succinylation and consequent impairment of tumor development both in vitro and in vivo [44].

In breast cancer, SIRT5 was shown to reduce ATP levels, thus rendering cancer cells more vulnerable to chemotherapeutic agents and environmental stress [69]. By desuccinylating and inhibiting GLS, an enzyme involved in the hydrolysis of glutamine into glutamate, and the subsequent formation of ammonia as a byproduct, SIRT5 contributes to the detoxification of ammonia [69]. In line with this, low levels of ammonia were discovered in MDA-MB-231 and C2C12 breast cancer cells overexpressing SIRT5, which reduced ammonia-induced autophagy and mitophagy [69]. This defense system protects against chemotherapeutic or stress-related processes such as hypoxia or starvation. Moreover, the glutamine catabolic byproduct α-KG, is essential for the anaplerotic replenishment of the TCA cycle and promotes ATP and lipid synthesis, which are essential for cancer cell proliferation. Hence, by interfering with these pathways, SIRT5 inhibits the growth of tumor cells [69].

SIRT5 also seems to function as a tumor suppressor in HCC and its expression was found to be reduced in primary liver cancer tissues compared with healthy liver tissues [119]. Particularly, aberrant activity of ACOX1, a peroxisomal enzyme involved in the formation of H_2_O_2_, results in oxidative damage to DNA and changes in FAO that affect liver function and ultimately lead to the onset of HCC. SIRT5 desuccinylates and inhibits ACOX1, which results in lower H_2_O_2_ levels and oxidative stress [63].

Lung cancer appears to be largely influenced by SIRT5. According to Lin and colleagues, succinylation of SOD1 causes a rise in lung cancer cell proliferation. Conversely, SIRT5-mediated desuccinylation activates SOD1, thus promoting ROS detoxification. Accordingly, rates of growth and replication of cells expressing a succinylation-resistant SOD1 mutant were reduced, thus correlating with the protective function of SIRT5 in this situation [46]. Additionally, SIRT5 is downregulated in NSCLC A549 cells. This causes STAT3 to be acetylated and translocated into the mitochondria, where it interacts with the PDC and stimulates the conversion of pyruvate to acetyl-CoA, thus promoting ATP synthesis and supporting cell growth [82].

SIRT5 expression is also reduced in androgen-independent PC-3 and PC-3M prostate cancer cells, with larger reductions occurring in more advanced stages of the disease. In line with this, studies using SIRT-KO PC-3 cells have revealed an increase in cell invasion, migration, and proliferation. In addition, the use of a peptide-based SIRT5 inhibitor (see compound **8d**, Section 4.2.2) highlighted the tumor suppressor function of SIRT5 by increasing PC-3 cell migration and invasion. The inactivation of lactate dehydrogenase A (LDHA) because of desuccinylation on Lys118 is hypothesized to be the cause of the tumor suppressor function of SIRT5. However, no mechanistic understanding of the part played by LDHA in the development and spread of prostate cancer was provided in the study [144].

### 3.7. SARS-CoV-2 Infection

Biochemical and cellular studies demonstrated that SIRT5 plays a significant role in the SARS-CoV-2 infection, even though two different studies point towards opposite conclusions. Indeed, while both reports agree that SIRT5 interacts with SARS-CoV-2 non-structural viral protein 14 (NSP14), they have opposite conclusions by suggesting that SIRT5 could act either as a proviral factor [145] or it could inhibit viral replication [146].

NSP14 is a 3′-5′ exoribonuclease and RNA-cap-guanine *N*7-methyltransferase, fundamental for viral replication [147,148,149]. Cellular thermal shift assay (CETSA) experiments found that when both SIRT5 and NSP14 are transfected into HEK293T cells, their stability is significantly increased. Moreover, the authors indicated that this interaction does not depend on NSP10, a protein partner of NSP14 interacting with its *N*-terminus. Interestingly, SIRT5 expression was also shown to be positively correlated with NSP14 expression [145]. The complete disappearance of binding between SIRT5 and NSP14 in studies using SIRT5 catalytic mutants that reduced its desuccinylase activity suggest that the catalytic activity of SIRT5 is required for interaction with NSP14. In line with this, when SIRT5-KD cells were transfected with NSP14 and SIRT5 and titrated with a SIRT5 inhibitor (see compound **8d** in Section 4.2.2) it was possible to observe a dose-dependent decrease in SIRT5–NSP14 interaction. Treatment of the same cells with the nicotinamide phosphoribosyltransferase (NAMPT) inhibitor F8866, which decreases NAD^+^ levels, also reduced the interaction, which was instead strengthened in the presence of nicotinamide mononucleotide (NMN), highlighting the importance of NAD^+^ co-accumulation and the necessity of SIRT5 catalytic activity for the interaction with NSP14. Although the catalytic activity of SIRT5 is required for binding to NSP14, none of the NSP14 lysine residues were found to be acetylated, succinylated, malonylated, or glutarylated and SIRT5 does not directly alter NSP14, hence NSP14 is not a target of SIRT5 [145]. Studies on SARS-CoV-2-infected A549-ACE2 cells in the presence of the specific SIRT5 inhibitor **8d** revealed a two-fold reduction in viral mRNA and a four-fold reduction in viral titers, whereas experiments on Calu-3 human lung cancer cells expressing ACE2 reported a two-fold reduction in both viral mRNA and viral titers. Additionally, a comparison of SIRT5-KO and WT A549-ACE2 cells infected with SARS-CoV-2 revealed a two-to-three-fold reduction in viral mRNA in SIRT5-KO cells. Overall, these data support the notion that SIRT5 operates as a proviral factor. In addition, since SIRT5 is involved in the RIG-1/MAVS antiviral pathway, an innate signal pathway that recognizes viral RNA in the cytosol and activates interferon type I [150], studies on MAVS-KO A549-ACE2 cells infected with SARS-CoV-2 in the presence and absence of **8d** have been conducted. MAVS-KO-infected cells exhibited three-to-five times greater viral levels both in the presence and in the absence of an SIRT5 inhibitor, thereby suggesting that SIRT5 activity is independent from the MAVS pathway [145]. Hence, SIRT5 seems to act as a proviral agent and the authors have provided several hypotheses. The first hypothesis is that NSP14 can enhance the activity of SIRT5, which would reduce the immune response and promote viral replication. A second hypothesis is that the NSP14/SIRT5 interaction could direct SIRT5 to new viral targets (NSP7, 8, 12, 13) involved in the complex replication transcription that may be deacylated by SIRT5, thus increasing their activity. Another possibility is that the NSP14/SIRT5 complex contributes to mRNA cap methylation, which renders viral RNA less detectable by the immune system [145].

A completely different perspective has emerged from a recent study by Liu and colleagues, who claim that SIRT5 inhibits viral growth due to desuccinylase activity that results from its interaction with NSP14 [146]. Experiments executed in CRC Caco-2 cells indicate that protein succinylation is particularly upregulated during the early stages of infection and is positively correlated with the period of viral infection [146], overall suggesting that this may be a host response during SARS-CoV-2 infection. The membrane glycoprotein (M) and nucleocapsid (N) proteins are succinylated ~24 h post-infection, demonstrating that succinylation occurs after translation during viral replication (between 12- and 24-h post-infection). Protein M has 2 succinylated sites, while protein N was succinylated in 12 sites, 2 of which (Lys65 and Lys102) are in the N dimerization domain, with the other 10 being close by and potentially influencing dimerization. Except for Lys65, which is unique to SARS-CoV-2, the rest of the N succinylation sites are conserved in bat SARS-like coronavirus and SARS-CoV, which also retain the two succinylated protein M sites. Since lysine residues are positively charged in the physiological environment, succinylation may have an impact on the protein N role in the SARS-CoV lifecycle [146]. Similar to the previous study, coimmunoprecipitation experiments revealed that SIRT5 can interact with NSP14. Through its interaction with SIRT5, NSP14 was indicated to increase the overall succinylation of host proteins. Additionally, in contrast to the previous study, experiments executed in Caco-2 and HEK293T-hACE2 cells revealed that SIRT5-mediated host protein desuccinylation may prevent viral propagation [146]. Proliferator-activated receptor γ coactivator 1-α (PGC-1α) and AMP-activated protein kinase (AMPK) have opposite effects on the SIRT5 expression level, with the first one stimulating its expression [151]. Hence, the authors assessed the influence of PGC1-α activators (valproic acid), AMPK inhibitors (GSK690693, ST1326, STO-609), and inhibitors of carnitine palmitoyltransferase 1A (CAPT1A), which has succinylase activity, on Caco-2 and HEK293T-hACE2 cells infected with SARS-CoV-2. The compounds exhibited antiviral activity to a certain extent, thus corroborating the hypothesis that SIRT5 desuccinylase activity contrasts SARS-CoV-2 replication. Nonetheless, it should be noted that some of the drugs employed are non-specific. For instance, valproic acid inhibits histone deacetylases and influences the expression levels of many proteins, including ACE2 [152,153]. Similarly, CPT1A inhibitors ST1326, glyburide, and etomoxir may target different proteins beyond CPT1A.

Overall, while both studies suggest that SIRT5 and NSP14 interact during SARS-CoV-2 infection, they end up with opposite conclusions regarding the role of SIRT5. These differences may be attributed to the different experimental approaches employed in the two studies such as the cell lines used for assessing the influence of SIRT5 expression on SARS-CoV-2 infection (A549-ACE2 and Calu-3 in the first study, Caco-2 and HEK293T-hACE2 in the second one). Hence, further studies would be necessary to clarify these discrepancies.

## 4. Pharmacological Modulation of SIRT5

Considering the key role of SIRT5 in a wide range of pathways, increasing research is being conducted over the possibility of targeting SIRT5 through either activators or inhibitors [47]. While the majority of studies have focused on SIRT5 inhibitors, recent reports have identified the first-in-class SIRT5 activators. This suggests that there is rising interest in developing both inhibitors and activators, paving the way to more specialized treatments and facilitating a better understanding of SIRT5 biological roles. SIRT5 activators will be examined in the following section, followed by an overview of the main SIRT5 inhibitors identified so far.

### 4.1. SIRT5 Activators

**Figure 5:**
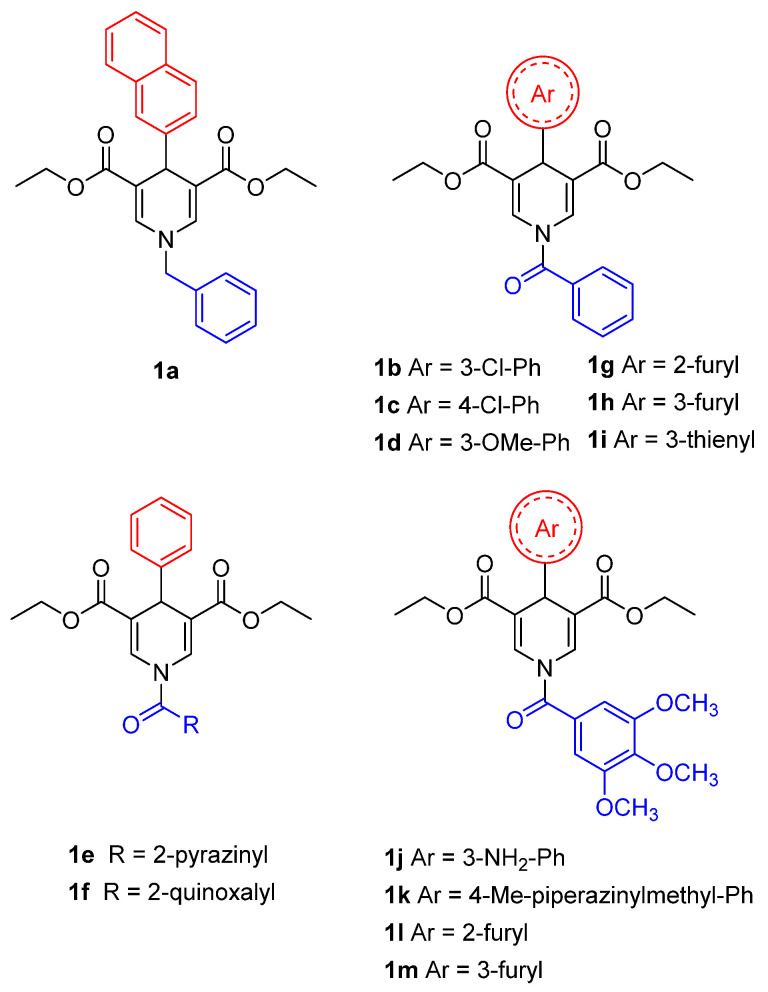
Structures of compounds **1a**–**1m**.

A recent study conducted by Suenkel and colleagues identified new 1,4-dihydropyridine (1,4-DHP) derivatives, bearing different substitutions at N1 and C4 (**1a**–**m**), as SIRT5-activating compounds (Figure 5) [154]. The first generation of compounds (**1a**–**f**) can increase SIRT5 desuccinylase activity two–three times at 100 μM and 1.2–1.5 times at 10 μM [154]. These compounds were tested for their influence on SIRT1-3 activity at both 10 and 100 µM, with only **1f** displaying two-fold activation of SIRT1 and SIRT3 at 100 µM. Overall, among this first series of derivatives, compound **1d**, bearing a benzoyl moiety at N1 and a 3-methoxyphenyl ring at C4, exhibited the best profile in terms of potency (>3-fold SIRT5 activation) and selectivity. The authors then prepared a second compound series, comprising compounds **1g**–**i**, presenting a dihydropyridine core with a benzoyl group on N1 and variously substituted rings on C4, and compounds **1j**–**1m** possessing a 3,4,5-trimethoxybenzoyl substituent on N1 and an (hetero)aromatic cycle at C4 (Figure 5). When tested at 100 µM, compounds **1g**, **1k**, **1l**, and **1m** exhibited a two-fold activation of SIRT5 desuccinylase activity, while **1h**, **1i** and **1j** increased SIRT5 activity by 3–3.5 times. In addition, compound **1i** demonstrated a five-fold rise in SIRT5 activity at 200 μM, an EC_50_ value of 40 μM, and a K_D_ of 28 µM. In terms of selectivity, when compounds were tested at 100 µM, **1h** could activate SIRT1 by 1.5-fold, and **1j**–**m** increased SIRT1 activity by two–three times. In addition, **1l** and **1m** exhibited 1.5-fold SIRT2 activation and **1j** could increase both SIRT2 and SIRT3 by two-fold. Overall, the only compounds of this series exhibiting selectivity for SIRT5 over SIRT1-3 were **1g** and **1i**. Compound **1i** was also tested against SIRT4 and SIRT6 at 100 μM and caused 50–60% inhibition of their deacylase activities, thus indicating a lower selectivity compared with **1g.** Additionally, when SIRT5 was titrated with NAD^+^ in the presence of compound **1i** (200 μM), it determined a sharp increase in its v_max_, highlighting that this activator acts by increasing SIRT5 substrate turnover. Furthermore, the addition of substrate peptide or NAD^+^ did not affect the K_D_ of **1i**, thus indicating that it binds to a pocket different from the active site and facilitates substrate turnover by inducing a conformational change.

Compounds **1d** and **1i** were then assessed in MDA-MB-231 breast cancer cells at 50 µM for 4 h and 24 h. In both cases, they could decrease the activity of GLS, a known SIRT5 substrate whose desuccinylation has been linked to reduced activity [69]. Consistent with this, lower ammonia levels were detected compared with control and GDH activity, which has glutamate (the product of GLS catalysis) as substrate. Similarly, when **1d** and **1i** were administered to PDAC cell lines S2-013 and Capan1 at 20 µM for 24 h they could both reduce the acetylation levels of SIRT5 substrate GOT1, while no effect was observed in mouse SIRT5-KO cells KPCS [154]. These results are in line with a recent report in which compound **1d** (MC3138) has been assessed in different PDAC cells lines [138]. Treatment with **1d** (10 µM, 24 h) produced a deacetylation profile akin to that brought on by SIRT5 overexpression, resulting in decreased GOT1 acetylation and inhibition of its enzymatic activity. Compound **1d** also reduced glutamine, glutathione, and pyrimidine metabolic pathway-related metabolite levels and impaired PDAC cell survival, with IC_50_ values ranging from 25.4 µM to 236.9 µM, while not being active in SIRT5-KO cells KPCS. Additionally, the combination of **1d** with the chemotherapeutic drug gemcitabine, a first-line therapeutic to treat PDAC patients, yielded synergistic effects at various dosages in human PDAC cell lines and patient-derived organoids. Moreover, administration of **1d** to patient-derived xenograft (PDX) mouse models of PDAC did not cause any significant alteration in body weight or blood biochemistry and its combination with gemcitabine decreased tumor volume, weight, and cell proliferation index [155].

### 4.2. SIRT5 Inhibitors

Research on SIRT5 inhibitors is still at its early stages, with few inhibitors confirming the activity in cellular studies. On the other hand, several peptide-based SIRT5 inhibitors have been described based on the crystallographic data on SIRT5 catalytic sites.

#### 4.2.1. Small Molecules

**Figure 6:**
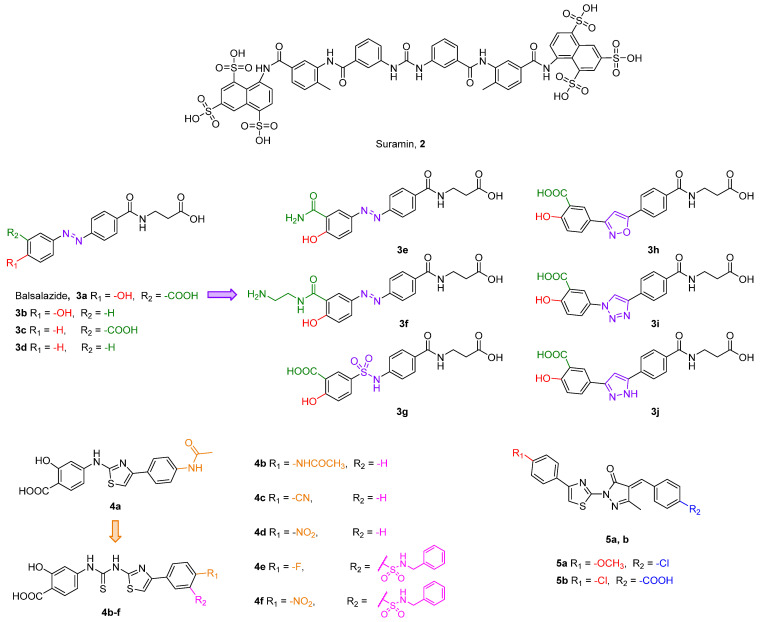
Structures of compounds **2**, **3a**–**j**, **4a**–**f**, and **5a**,**b**.

One of the first compounds identified as an SIRT5 inhibitor is the antiparasitic agent suramin (**2**, Figure 6). Compound **2** was shown to inhibit SIRT5 deacetylase activity with IC_50_ values between 14.2 and 26.8 μM [156,157,158] and SIRT5-mediated desuccinylation with an IC_50_ value of 46.6 μM [159]. Nevertheless, SIRT5 acts as a non-selective sirtuin inhibitor since it also inhibits SIRT1 and SIRT2 with IC_50_ values in the low-micromolar range [156,160]. The lack of specificity of compound **2** may be attributed to its binding mode. Indeed, the co-crystal structure of **2** bound to SIRT5 showed that it interacts with various residues in both the substrate and co-substrate binding sites [156]. As other SIRTs contain a similar co-substrate binding pocket, the fact that **2** binds to the NAD^+^ binding site may explain its lack of isoform specificity. Intriguingly, the authors also demonstrated that **2** causes SIRT5 dimerization in solution. Compound **2** forms multiple hydrogen bonds with the side chains of Tyr102, Arg105, and Tyr255, which are involved in substrate binding, as well as the backbone amide of Phe70 and the side chain of Arg71, which are all implicated in nicotinamide release. Finally, the carbonyl of the amide linked to the naphthalene moiety of **2** forms a hydrogen bond with the hydroxyl moiety of His158, thus replicating the interaction of the 3′-hydroxyl group of the co-substrate NAD^+^ [156].

Balsalazide (**3a**, IC_50_ = 3.9 μM, Figure 6) is a nonsteroidal anti-inflammatory drug presenting a salicylic group linked to a β-alanine-substituted benzamide via a central azo bridge initially identified as an SIRT5 inhibitor via a microchip electrophoresis-based screen [161]. Following this initial report, Glas and colleagues adopted **3a** as the lead compound in a SAR study in order to clarify its binding mode and to develop more potent compounds. They initially performed molecular docking based on an existing co-crystal structure of SIRT5 bound to a succinyl-lysine peptide in the presence of NAD^+^. They discovered that the carboxylate group of **3a** may form hydrogen bonds with Tyr102 and Arg105 and its amide moiety group may engage in further hydrogen bonds with Val221 and Glu225 backbones and the hydroxyl group of NAD^+^. This led them to conclude that the β-alanine moiety is primarily responsible for the inhibitory activity of **3a** [162]. Hence, they developed 13 analogues by deleting functional groups from the salicylic moiety, among which the phenol derivative **3b**, the benzoic acid **3c**, and the phenyl derivative **3d** were the most potent, although none of them outperformed **3a**. Indeed, when tested at 50 µM, compounds **3b**–**d** decreased the desuccinylase activity of SIRT5 by 73%, 63%, and 62%, respectively, compared with 83% inhibition exhibited by **3a**. The authors also measured the IC_50_ value of **3a**, which was 5.3 µM, in line with the previous study. When tested against different SIRT isoforms at 50 μM, compounds **3a**–**d** displayed no inhibitory activity, thus demonstrating SIRT5 isoform selectivity. Nonetheless, compound **3a** is barley soluble in water, has low oral bioavailability, and is subject to enzymatic hydrolysis [162]. Hence, with the aim of improving the unfavorable pharmacokinetic features of **3a**, Glass and colleagues set out to apply further modifications, which led to derivatives **3e**–**j**. In compounds **3e** and **3f**, the carboxylic group was replaced by a primary amide and an aminoethyl amide moiety, respectively. These compounds were tested at 50 µM against SIRT5 and decreased its activity by 87% and 80%, respectively, while **3a** exhibited 89% inhibition in the same assay. Further modifications were applied to the azo group, which was replaced by open-chained spacers, such as sulfonamide in **3g**, or five-membered heteroaromatic rings, including isoxazole (**3h**), 1,2,3-triazole (**3i**), and pyrazole (**3j**) (Figure 6) [163]. When tested at 50 µM, these compounds inhibited SIRT5 by 75% (**3g**), 80% (**3h**), or 84% in case of both **3i** and **3j**. Dose–response curves were also measured for **3g**, **3h**, **3i**, and **3j,** which displayed IC_50_ values of 12.5, 11.5, 7.4, and 7.7 μM, respectively, while **3a** showed an IC_50_ value of 13.8 µM in the same assay. Hence, replacing the azo group with sulfonamide and, in particular, heteroaromaic rings, is favorable for the inhibitory activity. Compounds **3a** and **3g**–**j** were also selective over SIRT1-3, while **3e** exhibited 27%, 41%, and 29% inhibition of SIRT1, 2, and 3, respectively, and **3f** was not tested for isoform-selectivity. Nonetheless, chemoproteomic experiments suggested that **3a**, **3i**, and **3j** bind to non-sirtuin off-targets, namely glutaryl-CoA-dehydrogenase (GCDH) and nucleoside diphosphate kinase (NME4), and exhibited EC_50_ values for binding in the dose-dependent responses in LC-MS/MS experiments in the same low-to-mid micromolar range. Live cell imaging assays in HeLa cells were employed to assess the influence of compounds **3a** and **3i** on the ability of SIRT5 to catalyze the formation of supramolecular fluorescent nanofibers. These assays indicated that compound **3i** could inhibit SIRT5 in cells at 250 µM, with a 90% decrease in fluorescent signal observed following 90 min of incubation. The same effect could be observed with **3a** only at 600 µM [164]. Overall, these experiments suggest that replacement of the azo group with the 1,2,3-triazole moiety increases the membrane permeability of this compound series [164].

Liu and colleagues recently reported another series of compounds bearing the same salicylic acid moiety as **3a** derivatives. Starting from the hit compound **4a** (Figure 6) identified through a thermal shift assay screening, the authors performed molecular docking-guided optimization. Enzyme inhibition studies indicated that **4a** inhibits SIRT5 with an IC_50_ value of 26.4 µM, while it was not active against SIRT1-3 even at 400 µM. According to docking results, the carboxylic acid forms key hydrogen bonds with Tyr102 and Arg105 side chains, while the benzene ring linked to the thiazole moiety forms π–π interactions with Tyr255. In order to maximize such interactions, numerous derivatives were synthesized, all of them possessing a thiourea group between the salicilic acid and thioazole moieties. Among them, compounds **4b**–**f** (Figure 6) were the most active, with IC_50_ values of 12.4, 11.4, 4.3, 8.2, and 2.5 µM. Interestingly, molecular docking suggested that, while the binding mode of **4b** and **4d** is analogous to that of **4a**, **4f** binds in a flipped position, with the nitro group forming a salt bridge with Tyr102 and Arg105 and the carboxylic moiety forming a hydrogen bond with Asn226. Selectivity studies indicated that **4b** and **4f** did not inhibit SIRT1-3, even at 400 µM.

Following a high-throughput screening of more than 5000 molecules, Yao and colleagues identified the hit compound **5a** bearing a E-4-benzylidene-5-methyl-2-(4-phenylthiazol-2-yl)-2,4-dihydro-3H-pyrazol-3-one scaffold (Figure 6), which inhibited SIRT5 with an IC_50_ value of 22.56 μM [158]. Molecular docking-guided optimization led to compound **5b** (Figure 6), which exhibited an inhibitory activity more than 100-fold greater than **5a** (IC_50_ = 0.21 μM), along with selectivity over SIRT1-3 and SIRT6 (up to 800 μM concentration). Docking experiments revealed that the thiazolyl moiety of **5b** forms π–π contacts with Tyr255 and the carboxylic group forms electrostatic and hydrogen bond interactions with Tyr102 and Arg105 in SIRT5 substrate-binding sites, while the carbonyl oxygen on the pyrazolone moiety forms a hydrogen bond with Arg71. Mechanistic studies revealed that the potency of **5b** is impacted by the presence of increasing succinyl-lysine substrate concentrations (IC_50_ with 30 μM of Ac-K(Suc)-AMC = 0.34 μM; IC_50_ with 270 μM of Ac-K(Suc)-AMC = 0.72 μM) but not by variations of NAD^+^ concentrations. These findings suggest that **5b** competes with the succinyl-lysine substrate, but not with NAD^+^, for its interaction with SIRT5 [158].

#### 4.2.2. Peptide-Based and Amino Acid Mimetics Molecules

**Figure 7:**
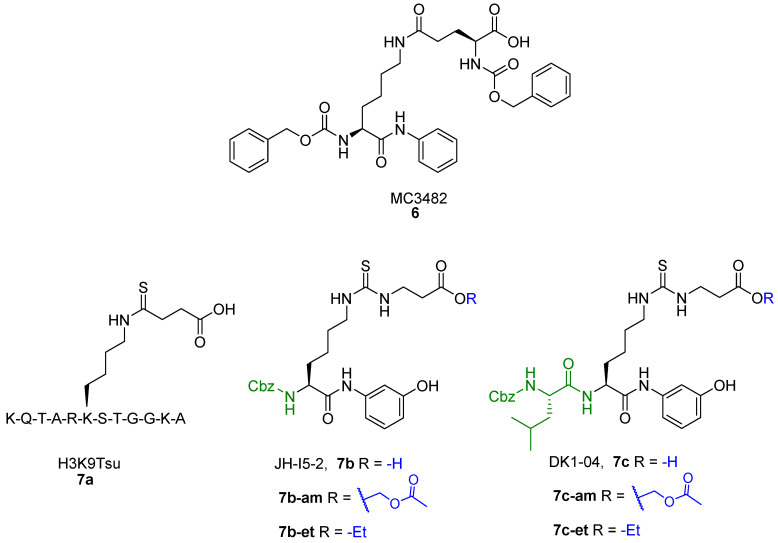
Structures of compounds **6**, **7a**, **7b**, **7b-am**, **7b-et**, **7c**, **7c-am**, and **7c-et**.

Recently, Polletta et al. developed MC3482 (**6**, Figure 7), a molecule based on ε-*N*-glutaryllysine possessing a benzyloxycarbonyl (Cbz)-protected amine on the lysine residue and an anilide function replacing the C-terminal carboxy [69]. When evaluated in MDA-MB-231 cells, compound **6** dose-dependently inhibited SIRT5-mediated desuccinylation, displaying 42% SIRT5 inhibition at 50 μM, while having no impact on SIRT1 and inhibiting SIRT3 by just 8% at the same dose. Furthermore, treatment with compound **6** (50 μM) led to a rise in succinylated proteins in both mouse myoblasts (C2C12) and human breast cancer cells (MDA-MB-231) due to the suppression of SIRT5 desuccinylase activity [69]. Additionally, compound **6** (50 μM) treatment of MDA-MB-231 and C2C12 cells increased GLS succinylation, which increased cellular glutamate and ammonia levels. These findings are consistent with the involvement of SIRT5 in controlling glutamine metabolism to regulate ammonia generation. Finally, **6** also enhanced ammonia-induced mitophagy and autophagy. Recently, compound **6** was also shown to be capable of stimulating the expression of brown adipose tissue markers when administered at an early stage of differentiation, thus enabling the differentiation of preadipocytes into brown-like adipocytes [165]. Furthermore, treatment with compound **6** at 50 μM increased mitochondrial activity and biogenesis, lipolysis rate, and the expression of triglyceride lipase. These results suggest that inhibiting SIRT5 may serve as an effective way to treat obesity and metabolic disorders [165].

Starting from a thiosuccinyllysine peptide (H3K9TSu, **7a**) that displayed a selective inhibition of SIRT5 desuccinylase activity (IC_50_ 5 µM; no SIRT1-3 inhibition at 100 µM), Abril and colleagues developed a series of peptidomimetic analogues, two of which exhibited promising SIRT5 inhibition [122]. Compound JH-I5-2 (**7b**) is a lysine derivative that presents *N*-terminal protection with a Cbz group and an *N*-(3-hydroxyphenyl) carboxamide group at the C-terminus and bearing a thiourea function instead of the thioamide moiety of **7a** (Figure 7) [122]. Even though the thiourea residue may be easily metabolized in vivo by cytochrome P450 and flavine monooxygenase (FMO) [166,167,168,169], producing hydrolysable sulfoxide derivatives to urea, compound **7b** demonstrated substantial inhibition against SIRT5 desuccinylase activity with an IC_50_ of 2.1 μM. Addition of a Cbz-protected Leu residue to **7b** *N*-terminus led to DK1-04 (**7c**), showing greater SIRT5 desuccinylation inhibition with an IC_50_ value of 0.34 µM (Figure 7). Both **7b** and **7c** were tested against SIRT1-3 and SIRT6 at 83.3 µM and did not affect their activity [122]. These compounds are mechanism-based inhibitors that disrupt the catalytic process by generating a covalent 1′-*S*-alkylimidate stalled intermediate with ADP-ribose within the catalytic site of SIRT5. To improve their cellular permeability, Abril and colleagues functionalized the carboxylic acid moiety with either aceto-methoxy (**am**) or ethyl ester (**et**). The resulting **7b-am**, **7b-et**, **7c-am**, and **7c-et** increased global lysine succinylation in MCF7 breast cancer cells at 50 μM. The viability of MCF7 and MDA-MB-231 breast cancer cells was considerably reduced by **7c**-based prodrugs (GI_50_ (**7c-am**) = 51 μM, GI_50_ (**7c-et**) = 20 μM). These also inhibited the anchorage independent growth of MCF7 and MDA-MB-231 cells with GI_50_ values ranging from 10 to 37 μM, with **7c**-based prodrugs being more effective. The most potent prodrug, **7c-et**, was administered at 50 mg/kg to both genetically modified (five times a week for 6 weeks) and xenograft (daily for 3 weeks) mouse models and could impair breast cancer growth in both cases [122].

**Figure 8:**
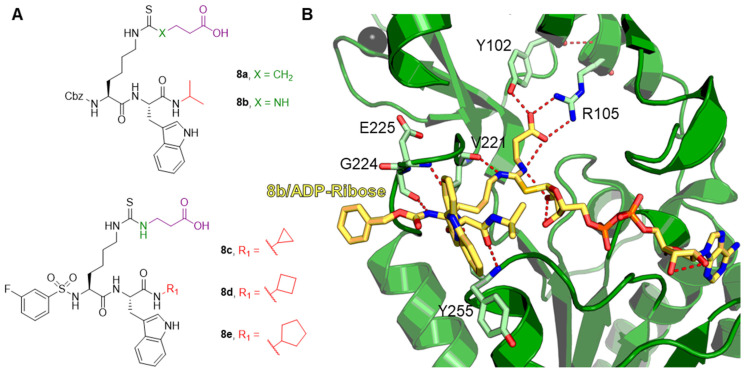
(**A**) Structures of compounds **8a**–**e**. (**B**) Focus on the binding site of hSIRT5 in complex with the ADP-ribose-1′-thioimidate intermediate of compound **8b** (yellow) (PDB ID 6EQS).

To clarify the chemical features required for SIRT5 inhibition, Rajabi and coworkers conducted a thorough SAR investigation by developing a variety of ε-N-thioglutaryllysine derivatives and testing them for the inhibition of SIRT5 deglutarylase activity [170]. Compound **8a**, carrying a thioamide moiety, a Cbz-protected *N*-terminus, and an *L*-Trp at the *C*-terminus, displayed an IC_50_ value of 0.83 μM [170]. Interestingly, compound **8b**, the thioureidic counterpart of compound **8a** (Figure 8A), displayed an IC_50_ value of 0.37 μM. The authors co-crystallized **8a** and **8b** with human SIRT5 and zebrafish SIRT5 and revealed the formation of a covalent catalytic intermediate with ADP-ribose and crucial interactions with Tyr102 and Arg105 (Figure 8B). Rajabi et al. continued the study by generating various compounds bearing different *N*- and *C*-termini. Among them, compound **8c** has a cyclopropyl at the *C*-terminus and a 3-fluorobenzensulfonamide at the *N*-terminus and showed an IC_50_ value of 0.26 μM. Further replacement of the *C*-terminal isopropyl with cyclobutyl (**8d**) or cyclopentyl (**8e**) resulted in IC_50_ values of 0.11 and 0.23 µM, respectively. Notably, **8d** was recently indicated to have an IC_50_ value of 0.44 µM against SIRT5-mediated desuccinylation [145]. These compounds function as mechanism-based inhibitors by facilitating the establishment of a covalent adduct with NAD^+^. Given their peculiar mode of action, the obtained IC_50_ values may not be compared with the ones attained with reversible inhibitors that are obtained with measurements at equilibrium. However, the *K_i_* values obtained for the most promising compounds from continuous flow tests allow a kinetic evaluation and a more precise calculation of the inhibitor efficacy. Specifically, **8a**, **8b**, and **8d** exhibit a slow tight-binding inhibition mechanism and their *K_i_* values are 22, 37, and 6 nM, respectively. Moreover, compounds **8b**–**e** were selective over SIRT1-3 and 6, while **8a** was not evaluated against other isoforms [170]. Compounds **8b** and **8d** were then assessed in cellular assays in the form of ester prodrugs (**8b-et** and **8d-et**), obtained by masking the negative charge of the carboxylic group with an ethyl ester in order to increase their cell permeability. AML cell lines with either SIRT5-dependent (OCI-AML2 and SKM-1) or SIRT5-independent (KG1a and Marimo) proliferation were examined. While **8b-et** and **8d-et** had no impact on SIRT5-independent AML cells, both molecules impaired cell growth and caused apoptosis in OCI-AML2 and SKM-1 cells. **8b-et** was the most potent compound, with IC_50_ values of 5–8 μM, roughly half of those measured for **8d-et.** Accordingly, **8b-et** triggered more than 80% apoptosis in SKM-1 (at 5 µM) or OCI-AML2 cells (at 10 μM), while **8d-et** could reach the same result only at 20 μM in SKM-1 cells. It is worth noticing that **8b-et** caused effects that were comparable to SIRT5 KD. Additionally, mice who received injections of **8b-et**-treated AML cells (at 12.5 or 25 μM) had better survival rates than the control group [135].

Notably, **8d** was also indicated to inhibit the interaction between SIRT5 and NSP14 (at concentrations from 25 µM onwards) in HEK-293 SIRT5-KD cells transfected with SIRT5 and NSP14. More significantly, SARS-CoV-2-infected Calu-3 cells showed decreased viral titers and mRNA levels when treated with **8d** at 25 and 100 μM, respectively [145].

**Figure 9:**
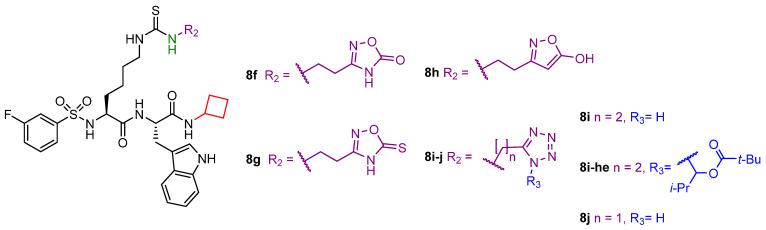
Structures of compounds **8f**–**j** and **8i-he**.

Rajabi and colleagues recently developed derivatives of **8d** to investigate if bioisosteric replacement of the carboxylic acid group could retain SIRT5 inhibition [136]. To this end, they prepared 1,2,4-oxadiazol-5(4H)-one (**8f**), 1,2,4-oxadiazol-5(4H)-thione (**8g**), 2-hydroxyisoxazole (**8h**), and tetrazole (**8i**) analogues of **8d** (Figure 9), which demonstrated submicromolar IC_50_ values against SIRT5 deglutarylase activity (IC_50_ (**8f**) ≤ 0.05 μM, IC_50_ (**8g**) = 0.9 μM, IC_50_ (**8h**) = 0.29 μM, and IC_50_ (**8i**) ≤ 0.05 μM). Additionally, the kinetics of how **8f**, **8h**, and **8i** inhibit SIRT5 were assessed. While **8g** had a K_i_ value of 122 nM, **8f** and **8i** displayed K_i_ values of 7 and 0.5 nM, respectively. All compounds were also selective over SIRT1-3,and 6, with only **8f** and **8h** showing some inhibition at 10 μM (38% SIRT1 inhibition and 40% SIRT3 inhibition, respectively). The significance of the length and flexibility of the lysine side chain for SIRT5 inhibition was demonstrated by the decreased potency of compound **8j** (IC_50_ = 5.1 μM), where the length of the alkyl spacer was decreased to a single methylene unit. Since these compounds displayed low cell permeability, the authors set out to mask the tetrazole group of **8i** with an O-tert-butyloxycarbonyl-N,O-isobutyl hemiaminal, thus leading to the prodrug **8i-he**. Notably, compound **8i-he** inhibited SIRT1 by 76% at 1 μM, while not having any effect on SIRT2, 3, and 6. Hence, in this case, the masking group reduced the isoform selectivity of the parent compound. The cellular target engagement of **8d**, **8d-et**, **8f**, **8g**, and **8i-he** was evaluated in HEK293T cells using an isothermal dose–response fingerprinting cellular thermal shift assay (ITDRF-CETSA). Compounds **8f** and **8g** demonstrated poor target engagement (EC_50_ > 10 μM), while **8d** and **8i** had EC_50_ values of 0.9 and 1.3 μM, respectively. Interestingly, **8d-et** and **8i-he**, carrying masked acidic groups, demonstrated the most significant target engagement (EC_50_ values of 0.25 μM and 0.15 μM, respectively). Moreover, **8i-he** was indicated to increase the melting temperature of SIRT1 and SIRT5, but not SIRT3, to a similar extent, thereby indicating only partial hydrolysis of the masking group inside HEK293T cells.

Compounds **8d**, **8f**, and **8i** were tested in HEK293T cells and SIRT5-dependent SKM-1 AML cells, where they could not decrease cell viability at doses up to 100 μM. In contrast, **8d-et** and **8i-he** showed IC_50_ values of 21 μM and 9 μM, respectively, against SKM-1 cells. When examined in HEK293T cells, **8d-et** exhibited an IC_50_ value between 50 and 100 μM, while **8i-he** demonstrated an IC_50_ > 100 μM with < 35% growth inhibition at 100 μM. This difference in IC_50_ values highlights that **8i**-**he** has higher cancer selectivity compared with **8d-et**. Finally, **8d-et** and **8i-he** were tested against OCI-AML2 and MOLM-13 SIRT5-dependent AML cell lines. Compound **8i-he** exhibited higher potency in OCI-AML2 cells (IC_50_ (OCI-AML2, **8d-et**) > 50 μM and IC_50_ (OCI-AML2, **8i-he**) = 20 μM), whereas comparable inhibition of cell growth was detected in MOLM-13 (IC_50_ (MOLM-13, **8d-et**) = 29 μM and IC_50_ (MOLM-13, **8i-he**) = 24 μM] [143].

**Figure 10:**
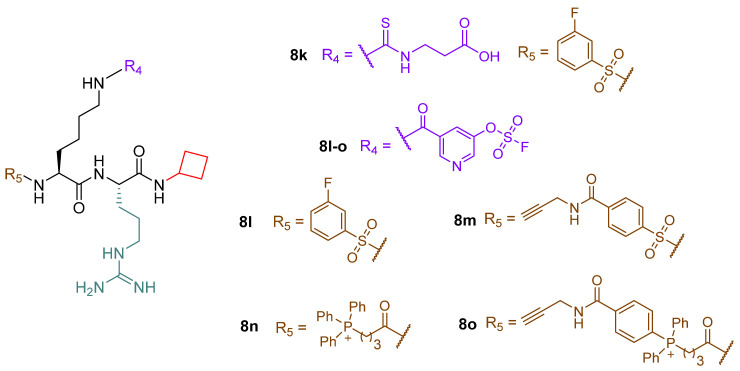
Structures of compounds **8k**–**o**.

The same group recently developed additional derivatives by introducing aryl fluorosulfate groups in order to obtain SIRT5 covalent inhibitors [171]. Starting from compound **8d**, different derivatives have been designed to enhance water solubility. This approach led to compound **8k**, an analogue of **8d** bearing a modified Arg residue at the C-terminus instead of Trp. Compound **8l** is a derivative of **8k** possessing a pyridin-3-yl fluorosulfate warhead replacing the carboxylic moiety, while **8m** has the same warhead as **8l** but contains a, N-terminal 4-(N-propargylcarboxamido)-benzenesulfonammide instead of the 3-fluorobenzenesulfonamide group. Additionally, compound **8n**, bearing a mitochondria-targeting triphenylphosphonium N-terminal moiety, and its N-propargylbenzamide-containing analogue **8o** were also prepared (Figure 10). These compounds were assessed for their influence on SIRT5-mediated deglutarylation at different incubation times (0–24 h). The parent compound **8k** did not exhibit great variation over time (IC_50_ (0 h) = 0.074 μM, IC_50_ (24 h) = 0.103 μM), while compounds **8k**-**o** exhibited time-dependent inhibition with IC_50_ values at 0 h over 200 µM, which decreased to 18–36 µM after 4 h and 4.6–6.1 µM after 24 h incubation (IC_50_ (**8l**) = 6.1 μM, IC_50_ (**8m**) = 5.0 μM, IC_50_ (**8n**) = 4.6 μM, IC_50_ (**8o**) = 4.9 μM) [171]. LC-MS analysis indicated that **8l**–**o** form covalent conjugates with SIRT5 and the presence of NAD^+^ was shown to increase the rate of covalent formation. Kinetic analysis performed on **8m** and **8o** provided K_i_ values of 98.4 and 139 µM, respectively. In-gel fluorescence imaging experiments also demonstrated that **8m** and **8o** could only form adducts with SIRT5, but not SIRT1-4, 6, or 7. Moreover, incubation of HEK293T cells overexpressing SIRT5-FLAG with compounds **8m** and **8o** (5 h at 20 µM) efficiently enabled SIRT5 pull-down following a click-chemistry reaction with azide-containing biotin and incubating the products with streptavidin-coated magnetic beads [171]. Furthermore, evaluation in HeLa cells, following the same approach described for compounds **3a** and **3i**, indicated that compounds **8l** and **8m** could decrease SIRT5 activity at 200 µM, with compound **8m** having a higher efficacy than compound **8l**. Compound **8l** was also assessed in different AML cell lines at 200 µM and had minimal influence on cell viability, with the exception of the Jurkat cell line, in which cell viability was almost abolished. The stability and bioavailability of compounds **8m** and **8o** were examined in mice by intravenous administration of a single dosage (12 mg/kg). For compound **8m**, this dosage was well tolerated (although with a mild sedative effect), while compound **8o** caused fast mortality, perhaps because of the triphenylphosphonium mitochondria-targeting group. In these trials, a quick fall in blood concentration and virtually full clearance within 5 min were observed. Nonetheless, after 6 or 24 h treatment with **8m**, Western blot analysis on mice cardiac tissue following **8m** conjugation with biotin indicated that SIRT5 was still labeled by **8m**. Hence, these results suggested that, despite its rapid clearance from the bloodstream, **8m** may still bind covalently to SIRT5 in mouse organs [171].

## 5. Conclusions

SIRT5 has been shown to recognize four different protein lysine modifications, thus suggesting that it probably exerts various biological effects depending on the presence of these modifications in various cell types or even in the same cell type in various physiological contexts. Through its catalytic activity, SIRT5 promotes glycolysis during metabolic reprogramming while inhibiting TCA cycling and electron transport. Furthermore, it is involved in FAO and ammonia detoxification processes [30,31,64,80,82,118]. By boosting NADPH production to assist GSH regeneration and by increasing SOD1 antioxidant activity, SIRT5 increases cell survival and lowers cellular ROS [46,64,80].

Like other SIRTs, SIRT5 has a double-faced role in cancer, acting as either a tumor promoter or suppressor not only in different types of tumors but also in the same ones under different experimental settings [52,70,72,114,119,120,147]. This suggests an intricate web of pathways regulated by SIRT5 activity, finally resulting in a context-dependent role affected by many factors, such as the specific mutation, cell and tissue type, and transformation stage. This is a result of the influence that SIRT5 has on redox homeostasis and ATP synthesis**.** Indeed, ROS neutralization contributes to DNA damage protection, which may be beneficial for keeping cells healthy on the one hand, but, on the other hand, this process protects cancer cells from apoptosis and promotes cell growth and resistance to genotoxic drugs.

Finally, SIRT5 activity was shown to be connected to the cellular response to the SARS-CoV-2 infection. Interestingly, two separate investigations reached contradictory findings regarding the proviral [145] or antiviral [146] activity of SIRT5 in this context. Nonetheless, both studies agree that SIRT5 interacts with the 3′-5′ exoribonuclease and RNA-cap-guanine *N*7-methyltransferase NSP14. Hence, further analyses would be necessary to further investigate the biological significance of this interaction and unravel the details of the involvement of SIRT5 in the SARS-CoV-2 infection.

SIRT5 is regarded as a potential therapeutic target for the treatment of different pathologies, including cancer, metabolic disorders, cardiovascular, and neurodegenerative diseases. Hence, it is vital to develop chemical probes that, by working as either activators or inhibitors, will contribute to clarifying SIRT5 biology. Further research on SIRT5 would also be beneficial for obtaining potent and selective modulators as potential therapeutics to treat those conditions in which SIRT5 plays a prominent role. In this context, the recent discovery of SIRT5 activators paves the way for the establishment of new strategies for facing SIRT5-related pathologies. In addition, the great amount of work that has been done on peptide-based inhibitors brings great promise for future optimization studies. To this end, the availability of co-crystal structures and high-throughput screening techniques would certainly facilitate the discovery of novel optimized inhibitors. For example, the co-crystal structure of SIRT5 in complexes with 8b/ADP-ribose would be crucial for designing new peptidomimetics with improved potency and pharmacokinetic properties.

Overall, gaining a deeper understanding of the activities of SIRT5 in various situations and elucidating the importance of its catalytic function in physiological and pathological conditions will be essential to further validate it as a therapeutic target. This is especially significant in cancer, because SIRT5 could serve as a tumor promoter or suppressor even in the same cancer subtype.

## Data Availability

Not applicable.

## Abbreviations

1,4-DHP	1,4-dihydropyridine
α-KG	α-ketoglutarate
ACAD	Acyl-CoA dehydrogenase
ACAT1	Acetyl-CoA acetyltransferase 1
ACOX1	Acyl-CoA oxidase 1
AD	Alzheimer’s disease
ADP	Adenosine diphosphate
AIFM1	Apoptosis inducing factor mitochondrion-associated 1
AML	Acute myeloid leukemia
AMP	Adenosine monophosphate
AMPK	AMP-activated protein kinase
ANXA1	Annexin A1
AP-1	Activator protein 1
ATP	Adenosine triphosphate
BAT	Brown adipose tissue
Bax	Bcl-2 associated X protein
Bcl-XL	B-cell lymphoma-extra large
cAMP	Cyclic adenosine monophosphate
CAPT1A	Carnitine palmitoyltransferase 1A
CoA	Coenzyme A
CDK2	Cyclin dependent kinase 2
CETSA	Cellular thermal shift assay
CPS1	Carbamoyl phosphate synthetase
CR	Caloric restriction
CRC	Colorectal cancer
CTL	Cell toxicity T lymphocytes
DRP1	Dynamin-related protein 1
EC_50_	Half maximal effective concentration
ECHA	Enoyl-CoA hydratase
EGFR	Epidermal growth factor receptor
ELOVL	Fatty acid elongase
ETC	Electron transport chain
FAD	Flavin adenine dinucleotide
FAO	Fatty acid oxidation
FFA	Free fatty acid
FOXO3A	Forkhead box O3
GAPDH	Glyceraldehyde 3-phosphate dehydrogenase
G6PD	Glucose-6-phosphate dehydrogenase G6PD
GLS	Glutaminase
GLUD1	Glutamate dehydrogenase 1
GOT1	Glutamic-oxaloacetic transaminase
GSH	Reduced glutathione
GSR	Glutathione-disulfide reductase
GSSG	Oxidized glutathione
HCC	Hepatocellular carcinoma
Hda1	Histone deacetylase 1
HDAC	Histone deacetylase
HMG	3-hydroxy-3-methyl-glutaryl
HMGCS2	3-hydroxy-3-methylglutaryl CoA synthase 2
HNSCC	Head and neck squamous cell carcinoma
IC_50_	Half maximal inhibitory concentration
IDD	Intervertebral disc degeneration
IDH	Isocitrate dehydrogenase
IFN-β	Interferon-β
IGF	Insulin-like growth factor
IL	Interleukin
iNOS	Inducible nitric oxide synthase
ITDRF-CETSA	Isothermal dose-response fingerprinting cellular thermal shift assay
KDAC	Lysine deacetylase
LC	Liquid chromatography
LCAD	Long-chain acyl-CoA dehydrogenase
LDHA	Lactate dehydrogenase A
LDHB	Lactate dehydrogenase B
LINC	Linker of nucleoskeleton and cytoskeleton
LPS	Lipopolysaccharide
MAPK	Mitogen-activated protein kinase
MCAD	Medium-chain acyl-CoA dehydrogenase
MITF	Microphthalmia family of transcription factors
MM	Multiple myeloma
MLS	Mitochondrial localization signal
MPTP	1-Methyl-4-phenyl-1,2,3,6-tetrahydropyridine
mRNA	Messenger RNA
MS	Mass spectrometry
NAD^+^	Nicotinamide adenine dinucleotide
NADH	Reduced nicotinamide adenine dinucleotide
NADP^+^	Nicotinamide adenine dinucleotide phosphate
NADPH	Reduced nicotinamide adenine dinucleotide phosphate
NAMPT	Nicotinamide phosphoribosyltransferase
NP	Nucleus pulposus
NRF2	Nuclear factor erythroid 2-related factor 2
NRF2/HO-1	Nuclear factor erythroid 2-related factor 2-heme oxygenase 1
NSCLC	Non-small cell lung cancer
NSP10	Non-structural viral protein 10
NSP14	Non-structural viral protein 14
OGDH	Oxoglutarate dehydrogenase
PARP1	Poly (ADP-ribose) polymerase 1
PD	Parkinson’s disease
PDAC	Pancreatic ductal adenocarcinoma
PDC	Pyruvate dehydrogenase complex
PDX	Patient-derived xenograft
PGC-1α	Proliferator-activated receptor γ coactivator 1-α
PKA	Protein kinase A
PKM2	Pyruvate kinase M2
PTEN	Phosphatase and tensin homolog
PTMs	Post-translational modifications
ROS	Reactive oxygen species
Rpd3	Reduced potassium dependency 3
SDH	Succinate dehydrogenase
SHMT2	Serine hydroxymethyltransferase 2
SIRT	Sirtuin
SOD	Superoxide dismutase
STAT3	Signal transducer and activator of transcription 3
SUN2	SAD1/UNC84 domain protein 2
TAC	Transverse aortic constriction
TCA	Tricarboxylic acids cycle
Th1	T-helper 1
THF	Tetrahydrofolate
TNF-α	Tumor necrosis factor α
TrxR2	Thioredoxin reductase 2
UCP-1	Uncoupling protein 1
VLCAD	Very long-chain acyl-CoA dehydrogenase
VDAC3	Voltage dependent anion channel 3
WAT	White adipose tissue
